# Venous-significant intracranial hemorrhage in neonatal HIE treated with therapeutic hypothermia: imaging phenotypes, diagnostic pitfalls, and prognostic implications

**DOI:** 10.3389/fnins.2026.1827015

**Published:** 2026-07-21

**Authors:** Linjie Hu, Jia Ye, Yaping Shen

**Affiliations:** Department of Pediatrics, Shengzhou People’s Hospital, Shengzhou Branch of the First Affiliated Hospital of Zhejiang University School of Medicine, The Shengzhou Hospital of Shaoxing University, Shengzhou, Zhejiang, China

**Keywords:** cerebral sinovenous thrombosis, hemorrhagic venous infarction, hypoxic–ischemic encephalopathy, intraventricular hemorrhage, MR venography, neonate, prognosis, susceptibility-weighted imaging

## Abstract

In the therapeutic hypothermia era, intracranial hemorrhage is increasingly detected in term and near-term neonates with hypoxic–ischemic encephalopathy (HIE), yet its clinical significance remains heterogeneous and is often oversimplified. This review uses a pattern-based approach for venous system–related intracranial hemorrhage in neonatal HIE, with particular emphasis on cerebral sinovenous thrombosis (CSVT) and hemorrhagic venous infarction. Magnetic resonance imaging plays a central role in etiologic assessment, with diffusion-weighted imaging and susceptibility-weighted imaging providing the core parenchymal and hemorrhagic information, while MR venography is most informative when imaging features raise suspicion for venous involvement. Imaging phenotypes that should prompt venous reconsideration include hemorrhagic parenchymal lesions crossing arterial territories and intraventricular hemorrhage accompanied by unilateral thalamic or deep parenchymal hemorrhage. Common pitfalls in neonatal MR venography interpretation–particularly flow-related artifacts–are also highlighted, emphasizing the importance of parenchymal–venographic concordance in avoiding both over- and underdiagnosis of CSVT. Mechanistically, hypoxia–reperfusion injury, endothelial activation, venous stasis, and developmental hemostatic vulnerability may create a milieu in which thrombosis and hemorrhage coexist. Available evidence suggests that long-term outcomes are driven more by associated venous-related parenchymal injury than by small-volume hemorrhage alone, underscoring the importance of pattern-guided imaging interpretation and follow-up.

## 1 Introduction

Neonatal encephalopathy (NE) remains a leading cause of mortality and long-term neurodevelopmental disability in term and near-term infants, with hypoxic–ischemic encephalopathy (HIE) accounting for the largest proportion (Chakkarapani et al., 2025; Zhao et al., 2026). Therapeutic hypothermia (TH) has become the standard of care for moderate to severe HIE and has substantially improved survival and overall neurological outcomes. Nevertheless, difficulties across multiple domains–including motor coordination, learning, language, and emotional/behavioral regulation–are still observed at school age, underscoring the importance of early risk stratification and long-term follow-up (Alqalyoobi et al., 2019; Chakkarapani et al., 2025; El-Dib et al., 2026).

Against this backdrop, the role of neuroimaging in HIE management has shifted earlier and gained greater weight. Contemporary imaging strategies for NE/HIE are MRI-centered (Cizmeci et al., 2025). Diffusion-weighted imaging (DWI) delineates patterns of hypoxic–ischemic injury, susceptibility-weighted imaging (SWI) identifies hemorrhagic burden while providing venous information, and proton magnetic resonance spectroscopy (^1^H-MRS) may be incorporated to strengthen prognostic assessment. In infants treated with TH, the optimal time window for DWI assessment typically falls after rewarming, around postnatal days 4–6 (Nagaraja, 2021). When clinical features or structural imaging raise concern for stroke or venous system involvement, adjunct arterial and venous imaging (MRA/MRV) facilitates etiologic classification and informs management decisions (Sacks et al., 2018).

Within this context of increasingly refined imaging, intracranial hemorrhage (ICH) is detected more frequently, yet its clinical significance is heterogeneous. MRI studies from historical HIE cohorts have shown that multiple hemorrhage subtypes may coexist with hypoxic–ischemic injury and are associated with more severe clinical encephalopathy and hemodynamic instability (Hillal et al., 2022; Saad et al., 2018). In contrast, studies in TH-treated populations suggest that although approximately one third of infants demonstrate ICH on MRI, hemorrhage *per se* does not exert an independent effect on neurodevelopmental outcome; rather, HIE-related MRI and MRS abnormalities remain the dominant prognostic determinants. These observations indicate that the simple distinction of “bleeding versus no bleeding” is not enough for risk stratification (Lakatos et al., 2019; Lally et al., 2019).

This review therefore centers on the working concept of venous system–related hemorrhage, with particular emphasis on phenotypes linked to cerebral sinovenous thrombosis (CSVT) and hemorrhagic venous infarction. CSVT in the neonatal period is clinically heterogeneous and prone to delayed recognition or underdiagnosis; when accompanied by parenchymal injury, it is associated with a substantially increased risk of adverse neurodevelopmental outcomes. Accordingly, identifying such hemorrhagic patterns in infants with HIE/NE not only helps explain why bleeding occurs, but also signals an underlying vascular etiology and additional injury burden, with direct implications for prognostic stratification and follow-up priorities.

Importantly, the wide variation in reported CSVT detection rates in TH-treated HIE/NE cohorts largely reflects differences in imaging practice. Across centers, substantial variability exists in whether MRV is routinely performed, in sequence selection, and in interpretive thresholds. In neonates, 2D time-of-flight MR venography (TOF-MRV) is particularly susceptible to frequent physiological and technical signal defects, including flow gaps and transverse sinus dominance. Interpreting these findings as thrombosis may lead to overdiagnosis, whereas dismissing them outright risks missed diagnoses.

Certain hemorrhagic patterns themselves serve as “red-flag” indicators of venous involvement. In term and near-term infants with intraventricular hemorrhage (IVH), CSVT is not uncommon; notably, the combination of IVH with unilateral thalamic hemorrhage should prompt heightened suspicion for venous system involvement and trigger venous imaging and etiologic reassessment.

Contributions of this review. Within the broader theme of HIE-associated intracranial hemorrhage, this review aims to: (i) propose a working definition and clarify the scope of venous system–related hemorrhage; (ii) synthesize current evidence on the spectrum, clinical recognition, and key imaging phenotypes of cerebral sinovenous thrombosis and hemorrhagic venous brain injury in the context of therapeutic hypothermia–treated HIE/NE, while discussing common pitfalls in MR venography interpretation; (iii) summarize available evidence regarding their implications for short-term complications and long-term neurodevelopmental outcomes, including epilepsy; and (iv) highlight priorities for future research, including more consistent imaging approaches, reporting frameworks, and outcome assessment to improve risk stratification and clinical counseling. The literature search approach and scope of the review are summarized in Box A.

BOX A **Literature search approach and scope.**

This narrative review was informed by targeted literature searches in MEDLINE, Embase, Web of Science Core Collection, and the Cochrane Library, supplemented by manual review of reference lists from key articles, reviews, and relevant guidelines. Searches focused on combinations of terms related to neonates/newborns, neonatal encephalopathy, hypoxic–ischemic encephalopathy, therapeutic hypothermia, cerebral sinovenous thrombosis, venous infarction, intracranial hemorrhage, MR venography, CT venography, and susceptibility-weighted imaging.

Priority was given to studies most relevant to the clinical questions addressed in this review, particularly those involving term or near-term infants, HIE/NE populations, venous imaging, hemorrhagic phenotypes suggestive of venous outflow impairment, and outcome data. We also included selected pediatric and neonatal CSVT studies, imaging methodology papers, and consensus/guideline documents where these were necessary to clarify diagnostic pitfalls, interpretive approaches, or management considerations.

Because the aim of this article is interpretive and clinically focused rather than quantitative, the review does not attempt formal study pooling or meta-analysis. Instead, the literature was used to develop a pattern-based approach for recognizing venous-significant hemorrhagic patterns in the setting of HIE, with particular attention to imaging concordance, MRV limitations, and prognostic implications.

## 2 Scope of the review and working definitions

To clarify this framework, MRI-detected intracranial hemorrhage in neonatal HIE may be stratified according to its likely venous significance rather than treated as a uniform finding.

### 2.1 Defining venous-related hemorrhagic phenotypes

Within the context of neonatal encephalopathy and hypoxic–ischemic encephalopathy, the spectrum of intracranial hemorrhage is broad, ranging from minor birth-related extra-axial bleeding to deep hemorrhages linked to vascular events and frequently accompanied by parenchymal injury (Chakkarapani et al., 2025; Cizmeci et al., 2025). Without first defining the hemorrhagic patterns of interest, highly prevalent but low-specificity findings–particularly extra-axial hemorrhage–together with uncertain attribution, in which vascular causes are grouped under the hypoxic–ischemic process, can make incidence, mechanism, and outcome data harder to interpret (Zhao et al., 2023). For clarity, this review adopts “venous system–related hemorrhage” as a working definition. This term refers not to a novel pathological category, but to a group of hemorrhagic phenotypes that are more likely to reflect impaired cerebral venous outflow and therefore be more informative for cause and prognosis in HIE.

From a practical standpoint, this definition prioritizes two clinical–imaging scenarios. The first comprises hemorrhagic venous infarction and its imaging-equivalent phenotypes associated with confirmed or highly suspected cerebral sinovenous thrombosis (Netteland et al., 2020; Son and Park, 2010). The second includes intraventricular hemorrhage in term or near-term infants accompanied by unilateral thalamic or other deep hemorrhage, a spatial configuration that strongly suggests venous outflow involvement (Abraham et al., 2023). As an extension, this review also considers white matter injury patterns that conform to venous drainage territories, particularly when susceptibility-weighted imaging demonstrates concordant venous signal abnormalities; however, the certainty of these patterns is considered lower than that of the core entities described above. By contrast, small-volume extra-axial hemorrhage, such as thin subdural or subarachnoid hemorrhage, is common and often self-limited in term neonates and is therefore treated here as background context, mainly relevant to epidemiological considerations or discussions of imaging sensitivity. Similarly, isolated hypointense segments or flow gaps on venous imaging, in the absence of concordant parenchymal injury, are not regarded as sufficient evidence of a venous event.

### 2.2 Core venous-significant hemorrhagic phenotypes

Within the working framework of venous system–related hemorrhage, two core phenotypes should be distinguished. They are related, but they are not interchangeable. CSVT-related hemorrhagic venous infarction represents established parenchymal injury linked to confirmed or strongly suspected venous outflow obstruction (Fitzgerald et al., 2006; Kersbergen et al., 2011a). By contrast, intraventricular hemorrhage accompanied by unilateral thalamic or thalamocaudate hemorrhage is best regarded as a deep hemorrhagic constellation that should trigger targeted evaluation for venous involvement in term and near-term infants (Lai et al., 2024; Wu et al., 2003).

#### 2.2.1 CSVT-related hemorrhagic venous infarction

This phenotype can be defined by three practical boundaries:

(1) Anatomical boundary: parenchymal injury located within a venous drainage territory, involving the dural venous sinuses, deep venous system, or major cortical venous drainage pathways.
(2) Imaging boundary: edema, restricted diffusion, venous-pattern infarction, and hemorrhagic transformation that are spatially concordant with venous outflow impairment.
(3) Clinical boundary: a high-information phenotype suggesting established venous-related tissue injury, rather than isolated hemorrhage or an isolated MRV signal defect.

Cerebral sinovenous thrombosis-related hemorrhagic venous infarction is the most direct phenotype of venous system–related hemorrhage. Its biological logic is straightforward. Thrombosis involving a dural venous sinus, the deep venous system, or less commonly a major cortical venous drainage pathway impairs venous return. Venous and capillary pressure rise, local perfusion falls, and the blood–brain barrier becomes vulnerable. The result may be edema, venous infarction, and hemorrhagic transformation. In this phenotype, the key finding is therefore not hemorrhage alone, but hemorrhage embedded within a parenchymal lesion that fits venous drainage anatomy (Fitzgerald et al., 2006; Kersbergen et al., 2011a; Lai et al., 2024).

The anatomical anchor is the drainage territory of the affected venous structure. Depending on the site of thrombosis, lesions may involve parasagittal cortex and subcortical white matter, temporal or occipital regions, deep gray nuclei, the thalami, or territories related to the deep venous system (Kersbergen et al., 2011b). Unlike arterial ischemic stroke, venous infarction often crosses arterial boundaries. This feature is particularly useful in neonates with presumed HIE, in whom global hypoxic–ischemic patterns may otherwise dominate the interpretation. Imaging clues include focal or regional swelling, restricted diffusion, vasogenic edema, hemorrhagic transformation, and susceptibility abnormalities that are spatially concordant with the suspected venous territory (Kersbergen et al., 2011a). When MRV, contrast-enhanced MRV, or CT venography demonstrates a matching venous abnormality, diagnostic confidence increases (Dlamini et al., 2010).

This boundary is important. CSVT-related hemorrhagic venous infarction should not be diagnosed from an isolated venographic signal defect. In neonates, two-dimensional TOF-MRV is sensitive to slow flow, acquisition plane, sinus caliber, skull molding, and normal transverse sinus asymmetry (Sarma et al., 2023; Widjaja et al., 2006). Segmental non-visualization, especially in the posterior superior sagittal sinus or transverse sinus, may therefore represent artifact rather than thrombosis (Pai et al., 2020). A venographic abnormality is most persuasive when it is supported by structural MRI, DWI, and SWI findings in the expected drainage territory. Conversely, if the parenchymal lesion strongly follows a venous pattern but TOF-MRV is equivocal, further adjudication with contrast-enhanced venography, phase-contrast venography, CT venography, or short-interval repeat imaging may be appropriate (Saposnik et al., 2024; Sarma et al., 2023).

The clinical significance of this phenotype lies in the associated parenchymal injury burden. Neonatal CSVT studies consistently suggest that outcome is shaped by the presence, location, and extent of brain injury, as well as by thrombus propagation and later epilepsy risk. Small-volume hemorrhage alone is a much weaker prognostic marker. For this reason, CSVT-related hemorrhagic venous infarction should be treated as a high-information phenotype: it identifies a vascular mechanism, indicates superimposed tissue injury, and supports more structured follow-up for motor, language, cognitive, behavioral, and seizure-related outcomes.

#### 2.2.2 Intraventricular hemorrhage with unilateral thalamic or other deep hemorrhage

This phenotype can be defined by three practical boundaries:

(1) Anatomical boundary: intraventricular hemorrhage accompanied by unilateral thalamic or thalamocaudate hemorrhage in a term or near-term infant.
(2) Imaging boundary: a deep hemorrhagic constellation that should prompt assessment for deep venous system, straight sinus, internal cerebral vein, or related venous drainage involvement.
(3) Clinical boundary: a red-flag pattern that triggers venous evaluation, but does not by itself prove completed venous infarction.

A second core phenotype is intraventricular hemorrhage in a term or near-term infant accompanied by unilateral thalamic or thalamocaudate hemorrhage. This phenotype overlaps mechanistically with CSVT-related hemorrhagic venous infarction, but its diagnostic role is different. It is not, by itself, proof of completed venous infarction. Rather, it is a recognizable deep hemorrhagic pattern that should prompt clinicians to evaluate the deep venous system, straight sinus, internal cerebral veins, and related venous drainage pathways (Kersbergen et al., 2009; Lai et al., 2024).

The strongest evidence concerns IVH with unilateral thalamic or thalamocaudate hemorrhage. In term infants, this combination is difficult to dismiss as a nonspecific background bleed. It differs from the classic germinal matrix hemorrhage pattern of prematurity and has been repeatedly linked to cerebral sinovenous thrombosis or deep venous outflow obstruction (Lai et al., 2024; Merlini et al., 2017). Similar concern may be reasonable when IVH is accompanied by anatomically plausible hemorrhage in adjacent deep gray matter structures, but this extension should be made cautiously (Lai et al., 2024). Alternative explanations–including prematurity-related germinal matrix hemorrhage, birth trauma, vascular malformation, and severe systemic coagulopathy–must be considered before assigning venous significance (Egesa et al., 2021).

This phenotype may first be detected on cranial ultrasound, conventional MRI, or SWI. Ultrasound may show IVH, thalamic echogenicity, or a deep hemorrhagic focus. MRI then helps define the distribution of hemorrhage, the presence of deep parenchymal injury, diffusion restriction, periventricular edema, and additional susceptibility findings (Kersbergen et al., 2009). SWI is particularly helpful for detecting small deep hemorrhages and venous-related susceptibility changes. In this setting, MRV is not being used as a universal screening test for every infant with HIE (Chakkarapani et al., 2025; Meoded et al., 2012). It is a targeted etiologic test prompted by a specific hemorrhagic pattern.

Clinically, IVH with unilateral thalamic or thalamocaudate hemorrhage should function as a red flag. When this pattern appears in a term or near-term infant with presumed HIE or neonatal encephalopathy, it should not be attributed automatically to therapeutic hypothermia, mild coagulopathy, or nonspecific perinatal bleeding (Lai et al., 2024; Merlini et al., 2017). A parallel or superimposed venous event should be reconsidered. The subsequent assessment should integrate structural MRI, DWI, SWI, venography, and the clinical course, including seizures, delayed deterioration, thrombocytopenia, dehydration, infection, and hemodynamic instability (Russ et al., 2021).

The prognostic interpretation again depends on tissue injury rather than ventricular blood alone. Small isolated IVH may have several causes and may not independently alter long-term risk. IVH accompanied by unilateral thalamic or thalamocaudate hemorrhage carries greater etiologic weight because it points toward deep venous drainage disturbance and possible CSVT. Risk should therefore be framed in terms of the extent of deep parenchymal injury, lesion evolution, venous recanalization or propagation, and later seizure and neurodevelopmental outcomes, rather than by the mere presence of IVH.

### 2.3 Deep medullary vein–related white matter injury as a lower-certainty clue

White matter injury related to the deep medullary veins is a supporting clue within the broader category of venous system–related hemorrhage. Periventricular white matter involvement may appear in linear, radial, or fan-shaped distributions and be accompanied by abnormalities of the deep medullary veins (Benninger et al., 2021). Even in the absence of dural sinus thrombosis, such patterns may reflect the impact of transient venous congestion or microthrombotic processes on white matter. Within this spectrum, susceptibility-weighted imaging is primarily valuable for checking whether lesions match venous physiological clues (Zedde and Pascarella, 2025).

At the same time, this phenotype is characterized by substantial heterogeneity, and the full cause often cannot be proven in an individual infant, so the finding should not be overextended. For these reasons, this review positions deep medullary vein–related white matter injury as a potentially coexisting mechanism and source of confounding, assigning it a lower certainty than cerebral sinovenous thrombosis–related hemorrhagic venous infarction and the combination of intraventricular hemorrhage with unilateral thalamic or other deep hemorrhage (Pin et al., 2023).

By contrast, small-volume extra-axial hemorrhage in term neonates has a high background incidence and a self-limited natural history, as demonstrated by longitudinal MRI studies (Babata et al., 2025). When discussed alongside venous parenchymal lesions, such hemorrhage can obscure findings that are more relevant to prognosis. In addition, structural pitfalls of neonatal venous imaging warrant explicit clarification at this stage. On 2D time-of-flight MR venography, so-called flow gaps are extremely common in the posterior segment of the superior sagittal sinus and in the transverse sinuses (Widjaja et al., 2006). Even when corresponding sinuses show normal opacification on CT venography, these signal defects remain frequent, indicating that they most often represent artifacts or slow flow rather than true thrombosis.

Accordingly, non-visualization of a venous segment on MR venography should not be equated with venous thrombosis. Interpretation must return to the concordance among source images, structural imaging, and susceptibility-weighted imaging, and to whether the overall phenotype conforms to venous drainage territory logic.

## 3 Incidence and composition of clinically relevant hemorrhage in HIE populations

### 3.1 Hemorrhage subtypes and nonspecific background findings in HIE cohorts

In imaging studies of term and near-term HIE, the mere fact that hemorrhage can be detected on MRI is not novel (Parmentier et al., 2022). The clinically useful question is how to distinguish high-background, low-specificity hemorrhage from patterns that point to a cause or add to parenchymal injury burden. If this distinction is not made upfront, subsequent discussions of mechanisms and prognosis are easily diluted by signals that are frequently detected but information-poor (Bashir et al., 2018).

The MRI-defined composition of hemorrhage types was dominated by subdural hemorrhage, followed by intraparenchymal hemorrhage, intraventricular hemorrhage, and subarachnoid hemorrhage. Hemorrhage frequency increased with greater clinical severity of HIE, yet did not show a linear relationship with thrombocytopenia or coagulation abnormalities. After multivariable adjustment, intraparenchymal hemorrhage was associated with the use of vasoactive or inotropic support. Interpreted in clinical context, these findings suggest that extra-axial hemorrhage, particularly subdural hemorrhage, is the most common but carries limited informational density, whereas hemorrhage types more likely to alter etiologic interpretation or indicate additional injury mechanisms cluster within less frequent entities such as intraparenchymal hemorrhage, intraventricular hemorrhage, and their associated venous events. Risk stratification based solely on the presence or absence of hemorrhage therefore tends to draw attention toward frequent but nonspecific extra-axial findings, while overlooking less common yet more explanatory venous-related phenotypes.

### 3.2 Incidence and implications of intraventricular hemorrhage in term and near-term HIE

Within term HIE populations, intraventricular hemorrhage is uncommon overall, and estimates are broadly consistent across study designs. Comparative cohorts evaluating hypothermia versus conventional care have reported low absolute rates, with a numerically higher proportion observed in hypothermia-treated infants. This observation, however, should not be interpreted as evidence that hypothermia causes intraventricular hemorrhage (Al Yazidi et al., 2014; Bashir et al., 2018; Gorelik et al., 2016; Vrselja et al., 2015). In these studies, the timing and intensity of cranial ultrasound surveillance differed substantially between treatment groups, with earlier and denser imaging in hypothermia-treated infants. Differences in imaging windows and monitoring intensity alone are sufficient to alter how often intraventricular hemorrhage is detected.

For the purposes of this review, the relevance of intraventricular hemorrhage lies less in whether it independently predicts worse outcome and more in what it may signal etiologically. In term and near-term infants, the occurrence of intraventricular hemorrhage–particularly when accompanied by unilateral thalamic or other deep hemorrhage–is more readily linked to impaired venous outflow, including cerebral sinovenous thrombosis. Such combinatorial phenotypes shift hemorrhage from a background finding to a pattern that warrants additional venous evaluation and etiologic reinterpretation. Their interpretation and their role as structured triggers within imaging pathways are addressed in greater detail in the imaging-focused sections that follow.

### 3.3 Sources of variability and comparability challenges in reported CSVT detection rates in TH-treated HIE cohorts

In contrast to the low-frequency but high-yield nature of intraventricular hemorrhage, reported rates of cerebral sinovenous thrombosis in TH-treated HIE populations vary widely (Deger et al., 2021). Some prospective single-center studies, using a strategy of routine venous imaging after rewarming, have reported detection rates exceeding one quarter of cases and have advocated broader incorporation of venography (Radicioni et al., 2017). At the other extreme, large retrospective cohorts that transitioned to routine venous imaging protocols have reported no diagnosed cases despite near-universal completion of venography, with only isolated cases identified in earlier phases when venography was performed selectively following suggestive structural findings (Munster et al., 2023).

These very different detection rates are more likely explained by differences in MRV technique, imaging timing, and interpretation than by true biological variation–particularly with respect to sequence choice, acquisition parameters, and interpretive thresholds in neonatal venous imaging (Rollins et al., 2005; Widjaja et al., 2006). Systematic evaluations of 2D time-of-flight venography in neonates without thrombosis demonstrate that apparent flow gaps are exceedingly common in major venous sinuses, especially in the posterior superior sagittal sinus and the transverse sinuses (Widjaja et al., 2006). Even when cross-referenced with CT venography, these segments often show normal contrast opacification, indicating that most such defects represent artifacts or physiologic slow flow rather than true thrombosis. From a pediatric imaging perspective, 2D time-of-flight techniques are sensitive to slow flow and in-plane saturation, making age-related artifacts frequent in infants (Chaturvedi et al., 2017; Widjaja et al., 2006).

It follows that when interpretation thresholds favor equating flow gaps with thrombosis, reported detection rates will rise; conversely, when greater weight is placed on source images, concordance with structural MRI and susceptibility-weighted imaging, or adjudication using contrast-enhanced three-dimensional venography or CT venography, detection rates decline substantially. Transforming the question of “how common is CSVT” from a debate of impressions into a problem of comparable data requires the establishment of reusable minimum standards: defining which phenotypes trigger venous imaging, specifying acquisition sequences and parameters, prioritizing source images over projection images for interpretation, clarifying when a second imaging modality is required for adjudication, and implementing centralized, blinded image review with consistency assessment. Only when these elements are explicitly embedded in protocols and executed across centers can incidence estimates of cerebral sinovenous thrombosis become more comparable and less dependent on local technique and reader preference. On this basis, the subsequent imaging and methods sections of this review propose a minimum reporting and interpretation set aimed at reducing the impact of detection-rate polarization on epidemiologic and prognostic inference.

## 4 Clinical clues for recognition

### 4.1 Overlapping and nonspecific symptom spectra

Recognizing cerebral sinovenous thrombosis within the clinical setting of neonatal encephalopathy and hypoxic–ischemic encephalopathy is challenging, largely because symptoms are nonspecific (Chen et al., 2008). Seizures, reduced responsiveness or lethargy, abnormal tone, feeding difficulties, and respiratory problems are common in critically ill neonates (McIntyre et al., 2021). These manifestations may reflect the course of hypoxic–ischemic injury, but they may equally represent outward expressions of impaired venous outflow (Busl and Greer, 2010). Prior syntheses have emphasized that signs of venous thrombosis are often subtle and readily “masked” by other disease processes (Chiewvit et al., 2011). Accordingly, bedside clinical clues are more useful for signaling the need to reopen etiologic reasoning than for providing immediate diagnostic categorization.

Retrospective cohorts provide quantitative context for this nonspecificity. Analyses of neonatal venous thrombosis have shown seizures to be the most common presenting feature, frequently accompanied by respiratory compromise, feeding difficulties, lethargy, hypotonia, or thrombocytopenia (Bhatt and Chan, 2021). Importantly, presentation is rarely monosymptomatic; instead, most infants exhibit combinations of symptoms (Erickson et al., 2021). Earlier reviews have further highlighted temporal heterogeneity: cases presenting within the first 48 h of life often coexist with perinatal stressors such as respiratory distress, fetal compromise, or asphyxia, whereas later presentations tend to be dominated by neurological features including seizures, lethargy, apnea, and feeding deterioration (Drapkin et al., 2019; Erickson et al., 2021; Opiyo and English, 2011).

When these observations are placed back into the HIE context, a key but easily overlooked point emerges: a clinical picture that closely resembles HIE does not exclude venous thrombosis. In the therapeutic hypothermia era, the imperative to initiate treatment within hours of birth necessitates early classification as “possible HIE.” This early classification is a treatment-enabling step, not the end of etiologic evaluation. Even when that diagnosis is appropriate, additional parallel factors may contribute to the encephalopathic presentation. Venous thrombosis is one such etiology that warrants consideration alongside HIE rather than outside it. However, this statement should not be interpreted as a recommendation to obtain MRI or MRV before initiating therapeutic hypothermia, or to delay HIE diagnosis while waiting for venous imaging. The first hours after birth should remain focused on clinical eligibility assessment, cardiorespiratory stabilization, neuromonitoring where available, and timely initiation of therapeutic hypothermia within the accepted treatment window, generally within 6 h of birth (Lemyre and Chau, 2018).

Instead, the practical implication is heightened clinical vigilance during the cooling and rewarming period, followed by phenotype-driven etiologic reassessment. For most cooled infants, the main imaging decision point remains the standard post-rewarming MRI. MRV should be added when the clinical course, cranial ultrasound, structural MRI, DWI, or SWI raises suspicion for venous involvement, such as delayed or worsening seizures, unexpected neurological deterioration, IVH with unilateral thalamic or deep hemorrhage, or a hemorrhagic parenchymal lesion that does not conform to canonical HIE injury patterns. Earlier MRI/MRV should be reserved for selected cases in which bedside findings raise an urgent, management-changing question.

### 4.2 Clinical context and temporal cues: perinatal acidosis, delayed symptoms, and systemic illness

Rather than searching for specificity in isolated signs, more transferable clues often emerge from clinical context and timing (Kersbergen et al., 2011a). Large surveillance studies with MRI confirmation have shown that, compared with arterial ischemic stroke, infants with venous thrombosis more frequently exhibit evidence of perinatal acidosis and are more often systemically unwell. Generalized seizures and lethargy are common, and both symptom onset and diagnostic confirmation tend to occur at later postnatal ages (Lin and Liebeskind, 2016; Markus and de Leeuw, 2023). Within an HIE framework, these observations should not be interpreted as positioning acidosis as a diagnostic marker of venous thrombosis. Instead, they underscore that critical illness is not unique to HIE: perinatal asphyxia, infection or sepsis, dehydration, and circulatory instability may coexist, creating an environment in which thrombosis and venous outflow impairment are more likely (Mota-Rojas et al., 2022; Rahman et al., 2025).

This point matters clinically. When caring for an infant with perinatal asphyxia who has been provisionally classified as having HIE, anchoring bias becomes a real risk. Subsequent seizures, depressed mental status, or deterioration in feeding or respiration cannot always be safely absorbed into the expected HIE trajectory. In some cases, a better explanation may be an asphyxial background complicated by a superimposed venous event. Imaging in such scenarios often reveals more etiologically informative phenotypes, including deep hemorrhage, intraventricular hemorrhage with unilateral thalamic involvement, or hemorrhagic venous infarction. Compared with isolated symptoms, these temporal and contextual cues reduce the likelihood of misattribution and help prioritize subsequent imaging evaluation.

### 4.3 Temporal discordance and triggers for reassessment

In the setting of hypothermia-treated HIE, the emergence or worsening of neurological symptoms after rewarming or several days after birth carries diagnostic weight because it may indicate that the clinical course is no longer fully explained by the initial hypoxic–ischemic insult. This point should be understood as a trigger for ongoing etiologic reassessment, not as a recommendation to obtain MRI or MRV before the initial HIE diagnosis is made or before therapeutic hypothermia is initiated. The core manifestations of hypoxic–ischemic injury typically declare themselves early after birth. Although venous thrombosis can be present from birth, it is more often recognized at later postnatal ages (Scaravilli et al., 2012). Synthesized evidence indicates that venous thrombosis may mimic hypoxic–ischemic encephalopathy, but symptom onset generally occurs later, with a median around the third postnatal day, and is frequently accompanied by coagulation abnormalities or persistent thrombocytopenia (Lin et al., 2021). Common risk backgrounds include infection or sepsis and dehydration.

It is equally important to emphasize what temporal discordance does not imply. Delayed or evolving symptoms do not automatically indicate a vascular event. Hypoxic–ischemic injury itself undergoes secondary evolution, and the emphasis on electroencephalographic monitoring during hypothermia and rewarming reflects the continued vulnerability of this period to hypoxia-driven dysfunction (Ye et al., 2019). Thus, the conclusion of this section is not that seizures after rewarming should be presumed venous in origin. Rather, when symptom emergence or progression does not align temporally with the established hypoxic–ischemic explanation and is accompanied by systemic illness signals such as infection, dehydration, persistent thrombocytopenia, or coagulation abnormalities, stroke and venous thrombosis should be reconsidered as possible parallel or superimposed etiologies while standard HIE management continues.

The endpoint of such reassessment is not diagnosis by symptoms alone, but the decision of whether the imaging protocol should be expanded to answer a specific arterial or venous question (Jonas Kimchi et al., 2007; Kersbergen et al., 2011a). In practice, when ultrasound suggests intraventricular hemorrhage extending toward the thalamus or abnormal venous sinus flow, confirmation and adjudication should proceed with MRI incorporating venous imaging (Zhang et al., 2023). For most infants treated with therapeutic hypothermia, this decision is best made at the time of the standard post-rewarming MRI, when structural MRI, DWI, and SWI can be reviewed together and venography can be added if the phenotype is suspicious for venous involvement. When the spatial organization of structural imaging, diffusion changes, and susceptibility findings aligns with venous drainage logic, interpretation of venography becomes substantially more robust, allowing symptoms, clinical context, and imaging to converge into a coherent causal narrative. Earlier MRI/MRV should be reserved for selected cases in which bedside findings raise an urgent, management-changing question–for example, progressive intraventricular or deep hemorrhage, suspected thrombus propagation, or neurological deterioration that cannot be safely explained by the expected HIE course. In other cases, the appropriate response is heightened vigilance during cooling and rewarming, followed by phenotype-driven venous evaluation during post-rewarming MRI.

## 5 Neuroimaging phenotypes and interpretive strategy

**FIGURE 1:**
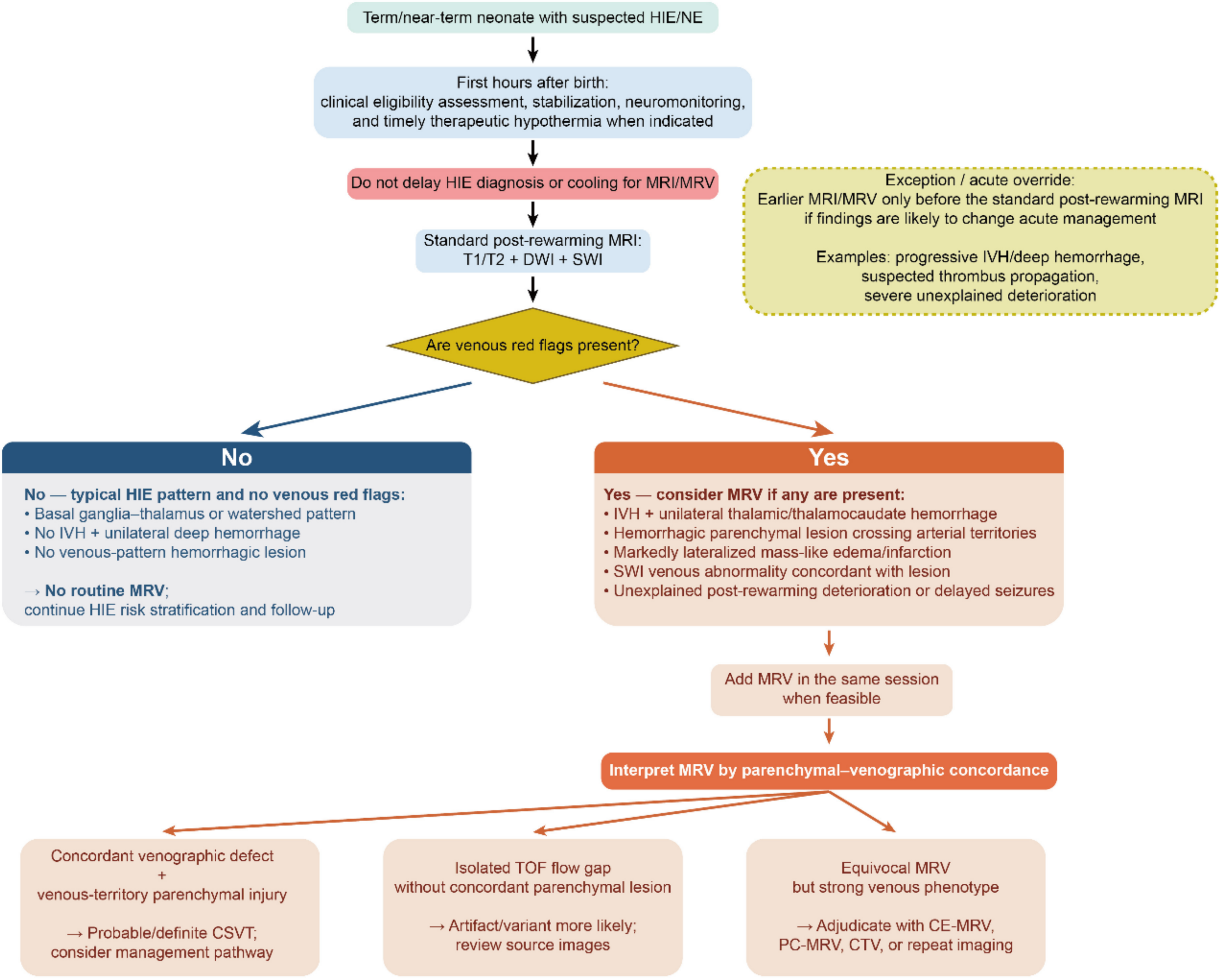
Phenotype-driven decision tree for adding MR venography in neonatal HIE/NE. A practical decision pathway for when to add MR venography in term or near-term infants with suspected HIE/NE. Early HIE diagnosis and therapeutic hypothermia should not be delayed for MRI/MRV. In most cooled infants, venous imaging is considered at the standard post-rewarming MRI when structural MRI, DWI, SWI, ultrasound, or the clinical course raises concern for venous involvement. MRV findings should be interpreted using source images and parenchymal–venographic concordance. Isolated TOF-MRV flow gaps favor artifact or anatomical variation, whereas concordant venographic defects with venous-territory parenchymal injury support probable or definite CSVT. Equivocal cases may require CE-MRV, PC-MRV, CTV, or repeat imaging.

When organizing image interpretation in the context of neonatal encephalopathy and hypoxic–ischemic encephalopathy, the primary task is not simply to determine whether injury is present, but to use a sufficiently comprehensive MRI dataset to complete etiologic screening within a single examination (Fiscone et al., 2024). This entails confirming hypoxic–ischemic injury while simultaneously seeking evidence of arterial or venous stroke, infection, structural abnormalities, and distinct hemorrhagic phenotypes (Adami et al., 2016). In practice, T1- and T2-weighted imaging, diffusion-weighted imaging, and susceptibility-weighted imaging constitute the core sequences, ideally with slice thickness ≤2 mm (Zhang et al., 2024). When clinical or imaging cues raise concern for arterial or venous system involvement, angiographic and venographic sequences should be incorporated in the same session. The true challenge lies not in deciding whether to add venography, but in reliably distinguishing between patterns consistent with “typical” hypoxic–ischemic injury and the lesions that follow venous drainage anatomy–while acknowledging that both processes may coexist in the same infant. The following sections therefore focus on reproducible starting points for image review, phenotype recognition, and common interpretive pitfalls. A practical phenotype-driven pathway for adding MRV–while preserving the priority of timely HIE diagnosis and therapeutic hypothermia (Figure 1).

### 5.1 “Match” and “mismatch” in structural MRI and diffusion patterns

The two canonical patterns of term and near-term hypoxic–ischemic encephalopathy–the basal ganglia–thalamus pattern and the watershed pattern–largely reflect the severity and temporal profile of the hypoxic–ischemic insult (Bobba et al., 2023). The basal ganglia–thalamus pattern is more often associated with abrupt and severe interruption of perfusion or oxygen delivery, involving deep gray matter and motor-related cortical structures, whereas the watershed pattern preferentially affects cortical and subcortical border zones and is more commonly associated with later profiles of relatively preserved motor function but impaired cognition or language (Van Cauter et al., 2020). Diffusion-weighted imaging is most sensitive for early recognition of these patterns; in hypothermia-treated infants, the optimal assessment window typically falls shortly after rewarming, around postnatal days 4–6 (Martinez-Heras et al., 2021). Toward the end of the first week, the risk of pseudonormalization increases, and during the second week lesion delineation relies more heavily on the temporal evolution seen on T1- and T2-weighted imaging (Oliphant et al., 1996).

**FIGURE 2:**
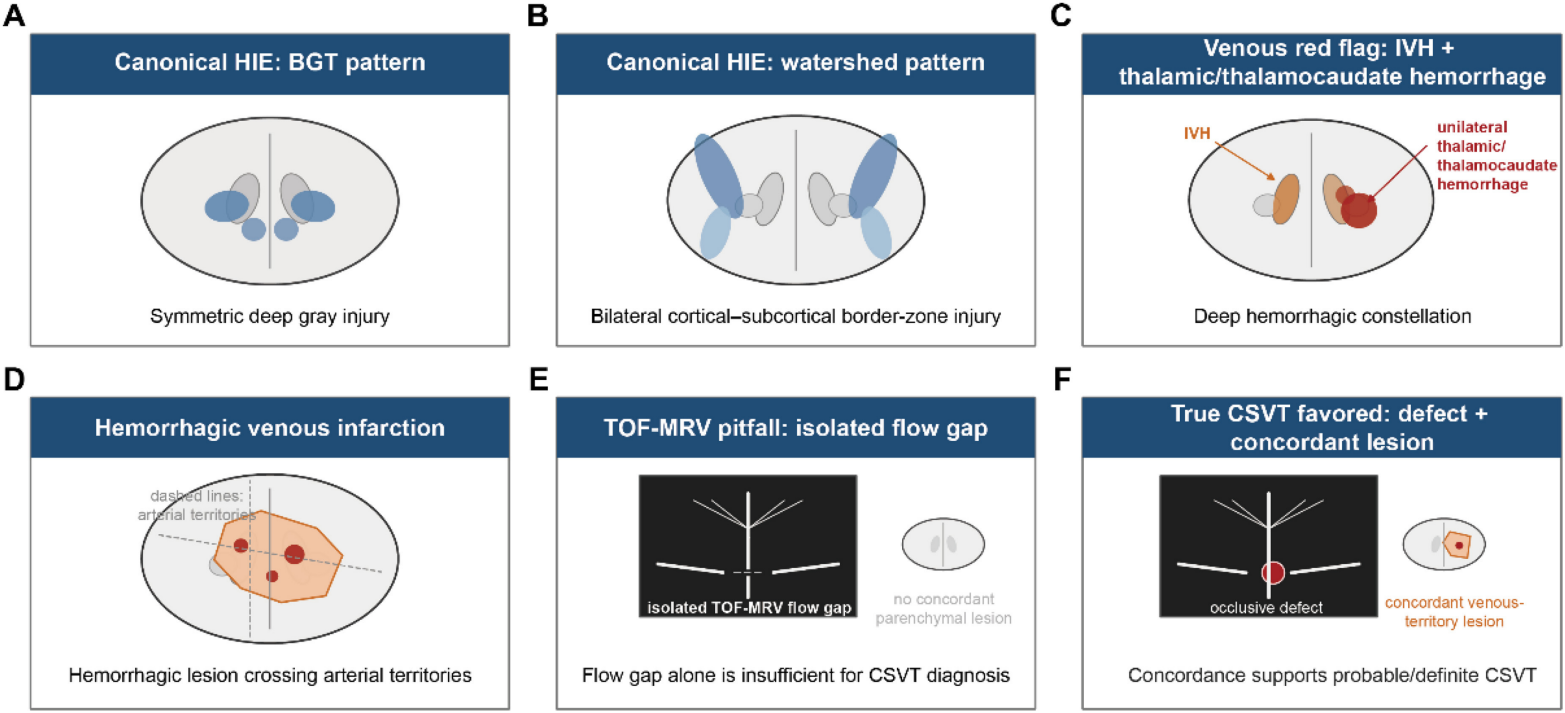
Schematic imaging phenotypes and MRV pitfalls in neonatal HIE/NE. Schematic teaching figure illustrating canonical HIE injury patterns, venous-significant hemorrhagic phenotypes, and common MRV interpretive pitfalls. This figure does not represent patient-derived imaging. **(A)** Basal ganglia–thalamus pattern of HIE, shown as symmetric deep gray injury. **(B)** Watershed pattern of HIE, shown as bilateral cortical–subcortical border-zone injury. **(C)** Venous red-flag phenotype: IVH with unilateral thalamic/thalamocaudate hemorrhage, a deep hemorrhagic constellation that should prompt evaluation for venous involvement in term or near-term infants. **(D)** Hemorrhagic venous infarction, shown as a hemorrhagic parenchymal lesion crossing arterial territory boundaries. **(E)** Common TOF-MRV pitfall: an isolated flow gap without a concordant parenchymal lesion. This pattern favors artifact or anatomical variation rather than CSVT. **(F)** Pattern favoring true CSVT: a venographic defect accompanied by a concordant venous-territory parenchymal lesion. This concordance supports probable or definite CSVT and may warrant confirmatory venous imaging when needed.

Using these temporal and spatial elements as a framework, the first interpretive question should be whether the observed abnormalities can be coherently explained by a global hypoxic–ischemic process (Yang et al., 2025). When they cannot, vigilance for venous events should be deliberately heightened (Nicolaides et al., 2024). The most common forms of “mismatch” arise from three spatial cues. The first is marked lateralization or asymmetry. Although hypoxic–ischemic injury need not be perfectly symmetric, lesions with strong unilateral predominance or mass-like edema or infarction that cannot be readily explained by global perfusion failure should prompt consideration of focal venous outflow impairment. The second cue is hemorrhagic parenchymal lesions that cross arterial territories. Venous infarction does not respect arterial boundaries and is more prone to hemorrhagic transformation; thus, the combination of hemorrhage, edema, and boundary-crossing distribution is more informative than hemorrhage alone (Babadjouni et al., 2017). The third cue is a specific constellation of deep structural involvement. In term and near-term infants, the presence of intraventricular hemorrhage accompanied by unilateral thalamic or other deep hemorrhage has repeatedly been associated with cerebral venous sinus thrombosis and aligns more closely with venous drainage logic than with basal ganglia–thalamus or watershed paradigms (Chang et al., 2025). These distinctions are summarized schematically by contrasting canonical HIE patterns with venous-significant hemorrhagic phenotypes (Figures 2A–D).

It is also important to recognize that some neonatal venous thromboses may recanalize early (Bhatt and Chan, 2021). Without timely venous imaging, diagnostic evidence may be erased by time itself, making attention to imaging windows and sequencing cadence critical (Dlamini et al., 2010). Accordingly, the role of structural imaging and diffusion-weighted imaging extends beyond severity scoring for hypoxic–ischemic encephalopathy. They should also be used as a pattern check: when lesions are symmetric, conform to established hypoxic–ischemic patterns, and lack venous cues, hypoxic–ischemic injury remains the most likely explanation; when one or more of the above mismatches are present, venous system–related hemorrhage should be advanced in the diagnostic hypothesis hierarchy and should guide subsequent sequence selection and interpretive focus (Park et al., 2022).

### 5.2 Susceptibility-weighted imaging: integrated depiction of hemorrhagic spectrum and venous clues

If diffusion-weighted imaging answers the question of where acute injury is occurring, susceptibility-weighted imaging and gradient-echo sequences address which blood products and venous information are embedded within that injury (Markl and Leupold, 2012). This functional distinction underlies the growing consensus that susceptibility-weighted imaging should be elevated to a core sequence in neonatal imaging protocols, alongside T1/T2-weighted imaging and diffusion-weighted imaging, with thin slices and the option to add angiographic or venographic sequences when stroke or venous thrombosis is suspected (Breutigam et al., 2022).

The added value of susceptibility-weighted imaging extends beyond hemorrhage detection. By virtue of its sensitivity to blood products and venous oxygenation differences, it can indirectly reflect changes in venous physiology and thereby increase the diagnostic sensitivity of MRI across a range of neonatal neurological conditions (Nitzan et al., 2020). Within the framework of venous system–related hemorrhage, susceptibility-weighted imaging provides at least three categories of information. First, it allows more sensitive and earlier characterization of the hemorrhagic spectrum, from small subependymal or periventricular foci to hemorrhagic venous infarction and deep nuclear hemorrhage accompanied by intraventricular extension, often enabling a more faithful assessment of hemorrhagic burden than conventional sequences (Meoded et al., 2012). Second, it offers indirect clues to altered venous filling and drainage. When venous outflow is impaired, local oxygen extraction increases and flow slows, cortical and medullary veins may appear more conspicuous on susceptibility-weighted imaging (Roy and Secomb, 2021). Such findings are not, in isolation, sufficient to diagnose venous thrombosis; however, when they align spatially with atypical parenchymal lesions–such as lateralized abnormalities, boundary-crossing distributions, or deep structural combinations–they substantially support a venous-event interpretation. Third, susceptibility-weighted imaging provides cross-validation for venographic interpretation (Haller et al., 2021). When time-of-flight venography suggests a potential defect, the presence or absence of concordant susceptibility signals, together with supportive structural changes, often reduces misclassification more effectively than reliance on venography alone.

Susceptibility-based techniques, however, carry intrinsic pitfalls. Their amplification of magnetic susceptibility differences produces blooming effects, whereby small hemorrhages or slow intravascular flow may be visually exaggerated. Moreover, the neonatal period is characterized by a background prevalence of minor hemorrhagic events (Velasco Gonzalez et al., 2025). Without calibration against structural imaging and clinical context, ubiquitous microhemorrhages risk being misinterpreted as etiologic turning points. For this reason, the optimal role of susceptibility-weighted imaging in this review is not as a standalone determinant of prognosis, but as a structured platform that integrates hemorrhagic morphology with venous physiological cues, informing whether venous imaging is warranted and how venographic findings should be interpreted.

### 5.3 When to add venography: phenotype-driven triggers

From the perspective of available evidence and consensus statements, venography is best understood as a problem-driven adjunct rather than a routine add-on applied indiscriminately to all cases of neonatal encephalopathy or hypoxic–ischemic encephalopathy (Satar et al., 2023). Authoritative reviews have consistently stated that angiographic and venographic sequences should be incorporated when arterial ischemic stroke or venous sinus thrombosis is suspected (Hinduja, 2025). Practice frameworks for neonatal brain MRI similarly recommend adding venography when there is concern for venous thrombosis, with or without associated parenchymal or intraventricular hemorrhage, while emphasizing the need for cautious interpretation given slow venous flow in neonates and the importance of appropriate sequence selection (Cochran et al., 2023). For most infants treated with therapeutic hypothermia, the practical decision point is the standard post-rewarming MRI, when structural imaging, DWI, and SWI can be interpreted together and venography can be added if the phenotype raises a specific venous question. The proposed decision pathway, including downstream interpretation of concordant and discordant MRV findings (Figure 1). Earlier MRI/MRV should be reserved for selected cases in which bedside findings or clinical deterioration raise an urgent management-changing concern.

Within the focused theme of venous system–related hemorrhage, the key issue is not whether venography should be mandated by protocol, but which clinical or imaging findings should trigger venography, and at what stage of care the venous question should be asked (Abdalkader et al., 2026). The most robust and reproducible trigger arises in the setting of intraventricular hemorrhage in term infants. Observational data have shown that a substantial proportion of such infants harbor cerebral venous sinus thrombosis, particularly when intraventricular hemorrhage is accompanied by thalamic hemorrhage (Neisewander et al., 2018). Subsequent syntheses have elevated this observation into a practical imaging cue: in term infants, intraventricular hemorrhage–especially when combined with unilateral thalamic or thalamocaudate involvement–should prompt strong suspicion of venous thrombosis. These phenotypes may also be accompanied by periventricular congestion, white matter changes distributed along medullary veins, and hemorrhagic lesions that may be missed on ultrasound.

In practical terms, venography should be considered when one or more of the following directional triggers are present:

(1) IVH in a term or near-term infant, especially when accompanied by unilateral thalamic or thalamocaudate hemorrhage;
(2) Hemorrhagic parenchymal lesions that cross arterial territories or do not fit basal ganglia–thalamus or watershed HIE patterns;
(3) Markedly lateralized, mass-like edema, infarction, or hemorrhagic transformation;
  Susceptibility-weighted imaging findings suggesting abnormal venous conspicuity or hemorrhage that is spatially concordant with a suspected venous drainage territory;
(4) Cranial ultrasound findings suggesting progressive IVH, deep hemorrhage, or abnormal venous sinus flow;
(5) Post-rewarming neurological deterioration or delayed/worsening seizures that are not well explained by the expected HIE course, particularly when accompanied by thrombocytopenia, coagulation abnormalities, dehydration, infection, or hemodynamic instability.

These triggers are intended to lower the threshold for MRV at the time of post-rewarming MRI, not to replace early HIE management.

This framework also clarifies why debate over the routine use of venography has persisted in neonatal encephalopathy cohorts. Many centers are not, in practice, comparing venography in unselected populations, but rather across differing degrees of problem-driven screening (Hedlund, 2013; Munster et al., 2023; Radicioni et al., 2017). This is exemplified by contrasting hypothermia-treated cohorts in which one strategy of routine post-rewarming venography reported high detection rates, whereas another large retrospective cohort found no diagnosed cases after venography was routinely incorporated, emphasizing instead the frequency of transverse sinus asymmetry as an anatomical variant and the sufficiency of conventional MRI to raise suspicion for venous thrombosis. These studies differ not only in whether venography was performed, but also in imaging timing, sequence selection, interpretive thresholds, and the weight assigned to parenchymal–venographic concordance.

Such contrasts do not support a binary conclusion that venography is either useless or mandatory. Instead, they redirect attention to the central methodological issue: when venography is highly dependent on technical implementation and interpretive thresholds, variability in detection rates is likely to reflect methodology rather than biology. This recognition motivates the focus of the subsequent section, which addresses how standardized acquisition, reporting, and interpretation frameworks can reduce the influence of detection-rate polarization on epidemiologic and prognostic inference. A phenotype-driven approach therefore preserves the urgency of early HIE treatment while ensuring that venous thrombosis is actively reconsidered when the clinical course or imaging pattern makes it plausible.

### 5.4 Common neonatal MRV artifacts and practical countermeasures

Understanding the pitfalls of neonatal MR venography is almost a prerequisite for understanding why reported detection rates of cerebral sinovenous thrombosis vary so widely across centers (Ayanzen et al., 2000). The practical reasons are straightforward. Two-dimensional time-of-flight venography has long been widely used in pediatric imaging because it is fast and does not require contrast administration. However, it is very sensitive to slow flow, in-plane flow, and complex flow patterns, and in neonates the boundary between anatomical variation and technical artifact is particularly blurred (Azarine et al., 2019). Reviews of pediatric venography have consistently emphasized that accurate interpretation of 2D time-of-flight studies requires familiarity with normal, age-related appearances; without this baseline, normal variants are easily misread as pathology.

Quantitative data show how common this problem is. Systematic evaluation of coronal 2D time-of-flight venography in neonates has shown that apparent flow gaps are extremely common: nearly seven in ten scans demonstrate such defects in the posterior segment of the superior sagittal sinus, more than half in the left transverse sinus, and approximately one quarter in the right transverse sinus. Importantly, when these same venous segments are assessed with CT venography, normal contrast opacification is typically present, indicating that the majority of flow gaps represent artifacts or physiologic slow flow rather than true thrombosis (Rollins et al., 2005; Widjaja et al., 2006). This distinction is particularly important when differentiating an isolated TOF-MRV flow gap from true CSVT supported by a concordant venous-territory lesion (Figures 2E, F). The underlying mechanisms align closely with neonatal vascular characteristics: smaller sinus calibers, lower flow velocities, and skull molding that can create focal narrowing (Zedde and Pascarella, 2025). In addition, when blood flow runs parallel to the acquisition plane, in-plane saturation may generate the illusion of signal discontinuity on projection images (Zanardo et al., 2021). Adjustments such as altering the acquisition plane or shortening the echo time can mitigate, but not eliminate, these effects. Common TOF-MRV pitfalls and practical cross-validation strategies are summarized for quick reference (Table 1).

**TABLE 1:** Common TOF-MRV pitfalls in neonates and practical cross-validation strategies.

Pitfall or interpretive issue	Typical appearance	Main risk	Practical cross-validation strategy
Segmental flow gap	Short-segment non-visualization, especially in the posterior superior sagittal sinus or transverse sinus	False-positive diagnosis of CSVT from a common neonatal TOF-MRV finding	Review source images; correlate with T1/T2, DWI, and SWI; do not diagnose CSVT from an isolated flow gap without a concordant venous-territory lesion
Transverse sinus asymmetry or hypoplasia	One transverse or sigmoid sinus appears smaller, faint, or poorly visualized	Normal venous asymmetry may be mistaken for thrombosis or occlusion	Assess continuity into the sigmoid sinus and jugular bulb; compare with structural MRI; use CE-MRV or CTV if uncertainty persists
Slow flow or in-plane saturation	Reduced or absent signal in venous segments with low velocity or flow parallel to the acquisition plane	Flow-related signal loss may mimic segmental occlusion	Consider acquisition geometry and hemodynamic status; use an alternative acquisition plane, PC-MRV, CE-MRV, CTV, or repeat imaging when clinically relevant
MIP-related overinterpretation	Apparent discontinuity or narrowing is prominent on MIP images but less convincing on source images	Projection images may exaggerate local signal loss, overlap, or partial-volume effects	Base interpretation primarily on source images; use MIP images for overview only; avoid a definite CSVT diagnosis from a single projection defect
Motion artifact or poor image quality	Blurring, ghosting, low signal-to-noise ratio, or apparent discontinuity across multiple venous segments	Both false-positive and false-negative MRV interpretation	Report image quality; repeat key sequences when feasible; avoid strong diagnostic conclusions from degraded MRV studies
Discordance between MRV and parenchymal imaging	Venographic defect without a matching lesion, or a strong venous-pattern lesion with negative/equivocal TOF-MRV	Overdiagnosis when MRV defects are isolated; underdiagnosis when TOF-MRV misses recanalized, deep venous, or small-vessel events	Apply parenchymal–venographic concordance as the central interpretive rule. If MRV is equivocal but the phenotype is strongly suspicious, adjudicate with CE-MRV, PC-MRV, CTV, or short-interval repeat imaging

These technical issues matter for hypothermia-treated encephalopathy research. First, when studies elevate time-of-flight defects or flow gaps to the status of primary diagnostic criteria for venous thrombosis, a high false-positive rate is almost unavoidable, particularly in the posterior superior sagittal sinus and transverse sinuses (Massrey et al., 2018). This inflates apparent detection rates and can create the impression, especially in small cohorts, that venous thrombosis is common. Second, even when venography is performed, inter-center differences in acquisition plane, echo time, slice thickness, reliance on source images versus projection images, and reader tolerance for transverse sinus asymmetry or dominance can push the same image toward diametrically opposed interpretations of “normal variant” or “thrombosis” (Kuo et al., 2019). Observations emphasizing transverse sinus asymmetry as a frequent anatomical variant serve as a reminder that, in the absence of shared interpretive standards, venography may reflect how images are read more than whether disease is truly present.

A more subtle but equally important source of bias operates in the opposite direction. Artifacts generate false positives, whereas time can generate false negatives. Partial or rapid recanalization may occur in some infants, such that if adequate venous imaging is not performed early, diagnostic evidence may be lost (Sullivan et al., 2022). Within hypothermia protocols, MRI is often scheduled after rewarming, which aligns well with the optimal diffusion-weighted imaging window for hypoxic–ischemic injury but may intersect imperfectly with the temporal evolution or resolution of venous events. Consequently, when detection rates across centers range from more than one quarter to nearly zero, the most coherent interpretation is not purely population-level variation, but the combined influence of venographic technique, sequence selection, timing, and interpretive thresholds (Han et al., 2023).

Taken together, the central message of this section can be distilled into a methodological reminder. In neonatal encephalopathy and hypoxic–ischemic encephalopathy, what most urgently requires standardization is not the binary decision of whether to perform venography, but rather the conditions under which a venous question is posed, the sequences used to answer it, and the interpretive thresholds by which the evidentiary chain is closed. Only when these elements are explicitly defined and applied consistently can venous system–related hemorrhage be transformed from imaging noise into a reproducible etiologic signal and an interpretable prognostic variable.

## 6 Pathophysiology: from systemic prothrombotic milieu to focal venous injury

Venous system–related hemorrhage is best explained by the coexistence of bleeding tendency and thrombosis risk in the same infant with neonatal encephalopathy or hypoxic–ischemic encephalopathy (Shen and Baker, 2015). On the one hand, laboratory findings may suggest a hypocoagulable state, such as prolongation of routine coagulation times or reductions in platelet count or fibrinogen (Danilatou and Zubair, 2025). On the other hand, neuroimaging may reveal distinctly “vascular” hemorrhagic phenotypes, including deep hemorrhage, intraventricular hemorrhage, or hemorrhagic venous infarction (Siddiqui et al., 2011). These observations are not contradictory. Hypoxia–ischemia, developmental hemostasis, and therapeutic hypothermia can create conditions in which thrombosis and bleeding coexist. The evidentiary strength across this chain is uneven: physiological and clinical data describing how the hemostatic system is altered are relatively robust, whereas links between these alterations and specific imaging phenotypes rely more heavily on cohort-level associations and pathophysiologically grounded inference (Phan et al., 2019). Accordingly, this section integrates systemic prothrombotic conditions and focal venous injury into a single causal framework, while explicitly defining the limits of inference.

### 6.1 Parallel activation of Virchow’s triad in the HIE/NE setting

Neonatal encephalopathy and hypoxic–ischemic encephalopathy are not single-organ disorders confined to the brain. Perinatal hypoxia followed by reperfusion can trigger a systemic inflammatory–coagulatory response characterized by endothelial activation, upregulation of tissue factor and procoagulant microparticles, and laboratory changes such as reduced fibrinogen, prolonged coagulation times, thrombocytopenia, and elevated fibrin degradation products, with some infants exhibiting a disseminated intravascular coagulation–like profile (Forman et al., 2014). Unlike adults, the neonatal hemostatic system represents a developmental network with low reserves but preserved compensatory capacity (Khizroeva et al., 2023). Coagulation factor levels and platelet reactivity are relatively low, whereas von Willebrand factor levels and hematocrit are relatively high, and natural anticoagulants are reduced (Kalot et al., 2022). The result is an apparent hypocoagulable baseline that can shift toward mild hypercoagulability under stress. Because buffering capacity is limited, systemic insults such as hypoxia, inflammation, or hemodynamic instability more readily tip the balance toward either thrombosis or bleeding.

Within the etiologic framework of cerebral venous sinus thrombosis, all three components of Virchow’s triad can be activated in parallel in this setting (Ranjan et al., 2023). The first is venous stasis. Circulatory instability following perinatal asphyxia, low cardiac output, volume fluctuations, and intensive care practices such as sedation and mechanical ventilation may all reduce intracranial venous flow velocity and pressure gradients. Although direct quantitative data are limited, this mechanism is physiologically plausible (Cramer et al., 2022). The second component is endothelial injury. Hypoxia–reperfusion induces endothelial activation and inflammatory–coagulatory coupling, often conceptualized as immunothrombosis, thereby increasing local thrombogenicity (Huertas et al., 2013). The third component is the coexistence of hypercoagulability and impaired fibrinolysis (Lee et al., 2023). Even when routine laboratory indices suggest hypocoagulability, viscoelastic testing in asphyxiated neonates has demonstrated reduced fibrinolytic potential, consistent with a state in which clot formation may occur but resolution is delayed.

Population-level evidence supports this construct. Large surveillance and nested case–control data show strong associations between hypoxia-related variables and neonatal venous sinus thrombosis, with effect sizes that remain substantial after multivariable adjustment. These findings indicate that the hypoxia–prothrombotic–venous thrombosis axis is supported by epidemiological data rather than resting solely on theoretical reasoning. From a mechanistic standpoint, it is therefore more accurate to state that neonatal encephalopathy and hypoxic–ischemic encephalopathy establish a background characterized by endothelial activation, altered flow, and suppressed fibrinolysis, within which venous thrombosis becomes more likely. Hypoxic–ischemic encephalopathy is not a sufficient cause of venous thrombosis, but it plausibly constitutes an important permissive environment.

### 6.2 Effects of therapeutic hypothermia on the hemostatic network: temperature, acidosis, and measurement mismatch

Therapeutic hypothermia is now standard care for moderate to severe neonatal encephalopathy and hypoxic–ischemic encephalopathy, and it also renders the fragility of hemostatic balance more visible (Lemyre and Chau, 2018). The clinically relevant question is not whether hypothermia affects coagulation, but to what extent these physiological effects translate into observable hemorrhagic or thrombotic events, and whether commonly used assessment tools accurately reflect *in vivo* hemostasis under hypothermic conditions.

From a mechanistic perspective, hypothermia exerts system-wide effects on the hemostatic network. First, enzymatic kinetics within the coagulation cascade slow, often manifesting as prolongation of routine coagulation times (Park and Park, 2024). These changes primarily reflect reduced reaction velocity rather than inevitable loss of clotting capacity. Second, hypothermia impairs platelet adhesion and aggregation, weakening primary hemostasis; this effect is typically rapidly reversible with rewarming (Wallner et al., 2022). Third, fibrinolytic and anticoagulant pathways may be altered in variable directions, depending on disease stage, acid–base status, and sampling conditions (Singh et al., 2023). Evidence from adult and mixed critical care populations suggests that mild to moderate hypothermia alone does not necessarily confer a major bleeding risk; however, when combined with moderate to severe acidosis, bleeding risk increases more consistently. Perinatal asphyxia represents a prototypical scenario in which hypothermia and acidosis coexist.

Neonatal cohort data provide bedside-relevant support for these mechanisms. During hypothermia, reversible impairment of platelet function can be observed, with rapid improvement after rewarming (Govindan and Loparo, 2024). Thrombocytopenia and hypotension are commonly reported adverse effects. However, equating laboratory abnormalities directly with clinical events is unreliable. Whether bleeding or thrombosis occurs depends on the interaction of systemic inflammatory–coagulatory coupling, circulatory stability, local hemodynamics, and tissue vulnerability.

A key challenge lies in the mismatch between assay temperature and patient temperature. Conventional coagulation tests are performed at 37°C, and thus reflect reaction behavior after rewarming rather than the *in vivo* state at 33°C–34°C. In contrast, viscoelastic testing can be performed closer to patient temperature, providing an integrated profile from clot formation through fibrinolysis. Within the same hypothermia-treated populations, parameters obtained at normothermic and hypothermic assay temperatures differ systematically, and some measures correlate with clinical bleeding. These observations underscore that temperature is a central explanatory variable in hemostatic assessment, not an ignorable source of noise.

Therapeutic hypothermia modifies coagulation and platelet function, but it does not by itself explain all hemorrhagic or thrombotic findings. It amplifies pre-existing hemostatic perturbations induced by perinatal asphyxia, while simultaneously increasing the risk of interpretive bias due to temperature-dependent measurement mismatch. For venous events, this bidirectional effect warrants particular caution. Prolonged routine coagulation times should not lead to automatic overestimation of bleeding risk, nor should they obscure the possibility of thrombosis (Schulman et al., 2024). Practical steps include explicit reporting of assay temperature, preferential use of viscoelastic testing at or near patient temperature, parallel interpretation of pre- and post-rewarming results with attention to directional change rather than isolated thresholds, and contextualization of laboratory findings within clinical status and imaging phenotype. When imaging features align with venous outflow impairment, mild to moderate laboratory abnormalities should not preclude evaluation for venous thrombosis; conversely, in the absence of supportive imaging phenotypes, isolated laboratory deviations should not be overinterpreted as evidence of clinically meaningful intracranial hemorrhage.

### 6.3 Why coagulation abnormalities alone do not explain key phenotypes: a more coherent causal organization?

If hypothermia-related coagulation abnormalities were the dominant mechanism, one would expect a monotonic relationship in which worse laboratory indices predict higher hemorrhage rates and specific intracranial hemorrhage phenotypes. Available data from hypoxic–ischemic encephalopathy cohorts do not support such a single-axis causal model.

On one hand, coagulation abnormalities are common and are associated with overall bleeding risk. Studies of moderate to severe hypoxic–ischemic encephalopathy report high rates of early hemostatic dysfunction, and abnormal bleeding events are not rare (Badulescu et al., 2025). Reduced fibrinogen and thrombocytopenia are associated with increased bleeding risk, and prospective data suggest that dynamic changes in coagulation parameters during the first postnatal week correlate with mortality and imaging abnormalities (Tamayo-Velasco et al., 2022). At a systemic level, the association between coagulation disturbance and bleeding is therefore real.

On the other hand, when analysis is confined to intracranial phenotypes, this association weakens substantially. Imaging studies demonstrate that subdural hemorrhage and some parenchymal hemorrhages are frequent but do not increase linearly with delivery mode, seizures, marked thrombocytopenia, or coagulation abnormalities (Liu et al., 2024). Instead, their frequency tracks more closely with overall disease severity. Analyses of hypothermia-treated cohorts similarly show that although intracranial hemorrhage is observed in a substantial minority, its mere presence does not independently determine neurodevelopmental outcome. Prognosis is shaped predominantly by the burden of hypoxic–ischemic injury on MRI and spectroscopy (Athiraman et al., 2020). Collectively, these findings suggest that many hemorrhages function more as accompanying phenomena than as decisive insults.

The critical distinction lies between two levels of causation: systemic bleeding tendency, described by fibrinogen levels, platelet quantity and function, and disseminated intravascular coagulation–like changes; and specific intracranial hemorrhagic phenotypes, which are more directly driven by hemodynamics and local vascular physiology (Zanza et al., 2023). This distinction is particularly important when the focus is venous system–related hemorrhage. Venous hemorrhage is typically not the result of failure to clot, but of failure of venous return. Impaired venous outflow elevates venous and capillary hydrostatic pressure, increases blood–brain barrier permeability, and reduces local perfusion pressure, thereby predisposing to hemorrhagic transformation in the setting of hypoxia–reperfusion (Esmon, 2009). Hemorrhagic venous infarction associated with venous sinus thrombosis exemplifies this chain and explains why deep hemorrhage or intraventricular hemorrhage can occur even when laboratory indices are not severely deranged (Katwal et al., 2023). Conversely, reliance on coagulation tests alone is insufficient to capture true vascular risk.

A more clinically and evidentially coherent framework therefore involves multiple linked steps. Neonatal encephalopathy and hypoxic–ischemic encephalopathy establish a systemic background in which prothrombotic and bleeding tendencies coexist, through endothelial activation, altered flow, and the low buffering capacity of developmental hemostasis. Therapeutic hypothermia further modifies coagulation and fibrinolysis dynamics and complicates assessment. Against this background, the occurrence or coexistence of venous sinus thrombosis becomes a pivotal node that shapes the emergence of clinically meaningful intracranial hemorrhage phenotypes and their prognostic implications. In practice, laboratory data should always be interpreted in conjunction with clinical context and imaging phenotype. When spatial patterns align with venous drainage logic–such as intraventricular hemorrhage with unilateral thalamic or deep hemorrhage, hemorrhagic parenchymal lesions crossing arterial territories, or markedly lateralized mass-like edema or infarction–venous mechanisms should be prioritized over attempts to explain all findings through isolated coagulation values alone.

## 7 Clinical management and follow-up

Re-embedding venous system–related hemorrhage into the real-world setting of neonatal encephalopathy and hypoxic–ischemic encephalopathy simultaneously reshapes the goals of imaging, the interpretation of hemorrhage, and the organization of follow-up. Imaging should not stop at phenotyping and grading hypoxic–ischemic injury; it should aim to complete an etiologic screen within the same MRI session (Parmentier et al., 2022). Hemorrhage should not be treated as a simple “more is worse” signal; instead, clinicians must distinguish phenotypes that merely record perinatal events and transient hemostatic perturbations from those that point to an additional vascular etiology and an incremental burden of parenchymal injury. Follow-up likewise should not be “closed” at discharge. Many risks emerge with developmental time, and long-term, cadence-based surveillance should be organized around motor, language, cognition/behavior, and seizure risk.

### 7.1 Imaging-driven decision nodes

Neonatal encephalopathy is a phenotypic syndrome rather than a diagnosis with a single etiology. Hypoxic–ischemic encephalopathy is the most common explanation, but it is not the only one (Russ et al., 2021). From this premise, MRI is best positioned as the first-line imaging modality, for etiologic clarification and prognostic assessment after the infant has been stabilized and the time-sensitive decision to initiate therapeutic hypothermia has been made. This statement does not imply that MRI should be obtained before HIE diagnosis or before cooling is started. In the first hours after birth, eligibility assessment for therapeutic hypothermia remains a clinical and time-critical process. Once the infant enters the standard neuroimaging window, MRI should include T1/T2-weighted imaging, diffusion-weighted imaging, and susceptibility-weighted imaging as the minimal core set with thin sections, and adding angiographic and venographic sequences when clinical or imaging features suggest a vascular process (Baliyan et al., 2016). In hypothermia-treated infants, diffusion-weighted imaging provides the most stable depiction of acute injury shortly after rewarming, around postnatal days 4–6 (Kasdorf et al., 2014). Thus, when new neurological symptoms emerge during rewarming or several days after birth, the imaging objective should shift toward an “etiologic MRI” that remains simultaneously sensitive to hypoxic–ischemic injury, arterial stroke, and venous outflow impairment. For most cooled infants, this means a phenotype-driven expansion of the standard post-rewarming MRI protocol, rather than a new routine early MRI/MRV pathway. Organizing care along these principles reduces the risk of missing key phenotypes because of suboptimal timing or sequence selection while preserving the urgency of early HIE treatment.

Completeness of workflow is necessary, but interpretability of conclusions is equally critical. The frequent artifact burden of neonatal time-of-flight venography can distort intuition about the “incidence” of venous thrombosis: flow gaps are common in the posterior superior sagittal sinus and transverse sinuses, and projection reconstructions may exaggerate the impression of discontinuity. Interpretation should therefore return to source images and prioritize concordance checks against susceptibility-weighted imaging and structural sequences (Ayanzen et al., 2000). When a suspected venographic defect lacks supportive ipsilateral parenchymal findings, it should be preferentially categorized as variant anatomy or technical limitation. Conversely, when parenchymal phenotypes conform to venous drainage logic but venography remains equivocal, adjudication with contrast-enhanced three-dimensional venography, phase-contrast venography, or, where feasible, CT venography may be warranted (Santucci et al., 2008). Rather than making venography a routine add-on for every infant, it is more defensible to treat it as phenotype-driven problem testing, with explicit documentation of the technical approach and interpretive thresholds so that venography adds information rather than noise. Earlier venous imaging should be considered only when the result is likely to change acute management, such as in progressive deep or intraventricular hemorrhage, suspected thrombus propagation, or neurological deterioration that cannot be explained by the expected HIE course.

These principles lead to a coherent set of practical priorities. Within constrained scan time, the first goal is to secure a minimal combination that maintains sensitivity to multiple etiologies. Susceptibility-weighted imaging should be treated as foundational rather than optional, because it can clarify both hemorrhagic burden and venous clues within the same dataset. Angiographic and venographic sequences should then be reserved for clinical and imaging scenarios that are genuinely directional–for example, intraventricular hemorrhage in a term or near-term infant accompanied by unilateral thalamic or other deep hemorrhage; hemorrhagic parenchymal lesions that cross arterial territories; or markedly lateralized, mass-like edema or infarction that cannot be reconciled with basal ganglia–thalamus or watershed paradigms. Additional triggers include SWI abnormalities that are spatially concordant with a venous drainage territory, cranial ultrasound findings suggesting abnormal venous sinus flow, and delayed or worsening post-rewarming seizures accompanied by systemic risk factors such as thrombocytopenia, dehydration, infection, or hemodynamic instability. Such integration preserves the central role of hypoxic–ischemic injury without allowing venous artifacts to mislead, and it enables symptoms, timing, and imaging phenotype to converge into a coherent causal chain practical diagnostic pathway that can guide downstream management and follow-up.

### 7.2 Current consensus and controversies in anticoagulation and supportive care

In the setting of neonatal encephalopathy and hypoxic–ischemic encephalopathy, management of venous system–related hemorrhage should begin not with the binary question of whether hemorrhage is present, but with the etiologic chain and the burden of tissue injury (Amer et al., 2023). When clinical and imaging features suggest venous sinus thrombosis–particularly when accompanied by venous infarction or deep hemorrhagic phenotypes–prevailing consensus supports active consideration of anticoagulation; small-volume, focal intracranial hemorrhage that aligns with venous pathophysiology is not an absolute contraindication (Lebas et al., 2012). The evidence base relies primarily on multicenter cohorts and expert consensus, and certainty is limited, but the directional signal is consistent: in pediatric venous sinus thrombosis, anticoagulation with heparin-based regimens is favored over observation alone, with the intent to limit thrombus propagation, promote recanalization, and reduce early adverse events.

Two considerations should be weighed together when deciding whether and when to start anticoagulation: evidence for venous thrombosis and active bleeding risk. The first is stronger when imaging shows a venous pattern. When imaging shows venous infarction, intraventricular hemorrhage accompanied by unilateral thalamic or other deep hemorrhage, or hemorrhagic parenchymal lesions crossing arterial territories, venous thrombosis should be elevated in priority and anticoagulation should be evaluated within that frame (Zhang et al., 2016). The second axis emphasizes tolerable bleeding risk. Uncontrolled active bleeding, large hematomas requiring surgical intervention, or uncorrected major hemostatic disturbances warrant stabilization, correction, and repeat imaging before entering an anticoagulation pathway (Bezati et al., 2025). These considerations should be assessed together rather than treated as separate decisions.

Treatment duration and reassessment are typically organized around combined imaging and functional monitoring. After stabilization, repeat MRI with venous imaging is performed within a predefined window to assess recanalization and thrombus progression, with adjudication by contrast-enhanced three-dimensional venography or CT venography when necessary (Smith and Hourihan, 2007). Clinical course, neurophysiological findings, and symptom endpoints are integrated to dynamically adjust intensity and duration. Across many centers, typical treatment spans weeks to months, followed by individualized de-escalation or extension based on recanalization status, residual risk factors, and recurrence risk; uniform evidence supporting long-term maintenance therapy is lacking (Rich et al., 2025). Supportive care determines whether anticoagulation can be delivered safely. Priorities include seizure control; correction of dehydration, infection, and electrolyte disturbances; maintenance of stable perfusion and ventilation; avoidance of positioning and care practices that compromise venous return; and coordinated management of platelet and fibrinogen thresholds with neonatal and hematology teams to prevent both thrombus propagation and hemorrhagic escalation (Hashmi and Wasay, 2011; Lebas et al., 2012). Family counseling should clearly distinguish goals: anticoagulation aims to limit thrombus progression and support recanalization, not to “remove hemorrhage.” It is also helpful to contextualize that small-volume background hemorrhage is common in hypothermia-treated cohorts and typically adds limited incremental prognostic information.

Current controversy centers on timing and treatment thresholds, particularly in the context of therapeutic hypothermia: whether to delay venous imaging until after rewarming, when diffusion findings and hemorrhagic stability may be clearer, or to intervene earlier once the diagnostic evidence is sufficiently compelling in order to limit thrombus propagation (Munster et al., 2023). Existing studies cannot fully disentangle confounding by illness severity, imaging timing, and venography methodology. A practical approach is to use two steps. First, entry into the pathway is defined by phenotypes with strong venous significance, reinforced by concordance between structural sequences and susceptibility-weighted imaging (Xue et al., 2025). Second, the hemorrhagic safety margin and systemic critical illness factors function as braking conditions, with repeated imaging and functional endpoints jointly anchoring efficacy and safety (Ferriero et al., 2019; Lebas et al., 2012). This framework helps avoid treating small-volume extra-axial hemorrhage as a contraindication while maximizing net benefit and minimizing risk when high-risk venous phenotypes are present.

### 7.3 Follow-up pathways: temporal dimensions of functional domains and seizure risk

Follow-up should extend beyond infancy because cognitive, language, behavioral, motor, and seizure-related difficulties may become apparent only later in development. Prospective follow-up of neonatal venous sinus thrombosis has shown that although recanalization often occurs within months, multi-year surveillance continues to identify substantial rates of neurological deficits and epilepsy (Bhatt and Chan, 2021). Longer follow-up increases detection, as children “grow into” their vulnerabilities, directly challenging the intuition that early normal findings guarantee long-term safety. Across the broader evidence base, despite heterogeneity in endpoints and thresholds, adverse outcomes occur in a substantial proportion of cases (Yakuwa et al., 2018). The presence and extent of parenchymal injury determine both the level and breadth of impairment, and seizure risk often becomes apparent later. These realities require shifting follow-up away from event-based indicators such as recanalization or residual hemorrhage and toward longitudinal evaluation of injury burden and functional trajectories, with core domains including sensorimotor function, language, cognition/behavior, and epilepsy.

Within the hypothermia-treated hypoxic–ischemic encephalopathy context, an additional calibration is required to avoid overburdening interpretation (Lemyre and Chau, 2018). Hemorrhage should not be uniformly equated with long-term catastrophe. Hypothermia-treated cohorts show that hemorrhage is detected in a substantial minority and is often small-volume; overall outcome is shaped predominantly by the burden of hypoxic–ischemic injury on MRI and spectroscopy, with hemorrhage itself not consistently demonstrating an independent effect (Lakatos et al., 2019; Lally et al., 2019; Rodrigues et al., 2025). When this is considered alongside the venous thrombosis literature, a clearer logic emerges: small-volume background hemorrhage alone is unlikely to reshape long-term risk narratives, whereas hemorrhagic phenotypes that suggest impaired venous outflow or indicate superimposed venous parenchymal injury warrant intensified and prolonged surveillance (Magid-Bernstein et al., 2022).

Follow-up can therefore be organized as a risk-oriented longitudinal narrative rather than a one-time discharge conclusion. In early infancy, emphasis should be placed on baseline neurological examination, head growth, tone, and gross motor milestones to establish the broad trajectory (Hadders-Algra, 2021). At 18–24 months, standardized developmental instruments can refine language and cognitive assessment, and active screening for epilepsy risk should be incorporated in high-risk phenotypes, including venous infarction, deep structure involvement, and intraventricular hemorrhage accompanied by unilateral thalamic hemorrhage. During the preschool and school-age periods, attention should shift toward later-emerging domains such as executive function, attention, and behavioral regulation, with neuropsychological assessment and educational planning when indicated (Faruk et al., 2020). New seizures, functional regression, or a history of substantial venous parenchymal injury should trigger repeat imaging and neurophysiological evaluation as a mechanism for re-stratification. This pathway embeds the time dimension into outcome interpretation: it does not merely record normality or abnormality at a single moment, but traces the slope and inflection points of functional development (Luo et al., 2018).

Ultimately, the value of follow-up lies in communicating and operationalizing three points. Neonatal encephalopathy and hypoxic–ischemic encephalopathy constitute a syndrome; imaging functions as an etiologic tool; and the prognostic meaning of hemorrhage is mediated chiefly through its indication of vascular etiology and its reflection of parenchymal injury burden. Outcomes do not end at discharge; they become legible over subsequent years. Organizing long-term follow-up around this logic avoids overinterpretation of small-volume hemorrhage while enabling earlier identification of modifiable risk windows introduced by venous injury.

## 8 Prognosis and risk stratification

In neonatal encephalopathy and hypoxic–ischemic encephalopathy populations, the prognostic meaning of “intracranial hemorrhage” is frequently misread in two opposite ways. One interpretation equates any hemorrhage with a worse outcome; the other dismisses hemorrhage wholesale as perinatal noise. What is genuinely useful for clinical counseling is to reframe the question from “is hemorrhage present” into two more discriminating dimensions: which etiologic chain the hemorrhage implicates, such as whether it signals a vascular event like cerebral venous sinus thrombosis, and whether a quantifiable burden of parenchymal injury has already been superimposed, including venous infarction, hemorrhagic venous infarction, deep structure involvement, or extensive edema. Together, these dimensions shape the outcomes families care about most: acute survival and complications, post-discharge neurological function and seizure risk, and later-emerging deficits that become apparent with development.

### 8.1 Overall outcomes after CSVT and the follow-up window

Prospective follow-up cohorts provide some of the most stable data for counseling anchors regarding the baseline risk profile of neonatal cerebral venous sinus thrombosis (Moharir et al., 2011). In a well-characterized prospective cohort of imaging-confirmed cases with standardized follow-up, acute-phase mortality attributable to venous thrombosis was present but relatively low, and thrombus propagation emerged as an important early event (Dlamini et al., 2010). Over multi-year follow-up, neurological abnormalities were observed in a substantial proportion of survivors, and epilepsy occurred at a frequency high enough to warrant anticipatory guidance and structured surveillance (Dlamini et al., 2010; Moharir et al., 2011). Importantly, these adverse outcomes were not limited to mild developmental delay. Impairments spanned multiple domains, including sensorimotor, language, and cognition/behavior, and moderate-to-severe disability was not uncommon.

These observations have immediate implications for how counseling should be framed. They correct the common intuition that “most infants do well after venous thrombosis” into a more accurate statement: early mortality risk is not dominant but is real; neurological problems among survivors are common; and seizure risk is clinically meaningful (Campillo et al., 2010; Moharir et al., 2011). A second, frequently underappreciated insight concerns the timing of prognostic certainty. Longer follow-up increases detection of adverse outcomes, because some deficits may not be apparent at discharge or in early infancy (Smith et al., 2013). Consequently, prognosis based on discharge status or a single early infancy assessment is systematically biased toward optimism.

Variation in outcomes across cohorts should not be dismissed as unreliability; rather, it should be interpreted as a signal of differences in case mix and diagnostic windows. Cohorts enriched for more severe illness or those in which diagnosis is preferentially made in symptomatic infants are expected to show higher mortality and higher rates of major sequelae. This heterogeneity reinforces a core point: prognosis after venous thrombosis is not a single number, but a distribution that shifts with case composition, complications, and imaging phenotype.

### 8.2 Parenchymal injury burden determines risk tiers

The quality of stratification ultimately depends on structured assessment of parenchymal involvement. Across syntheses and prospective data, adverse long-term outcomes occur in a substantial fraction of neonatal venous thrombosis cases, and they align strongly with whether parenchymal injury is present (Fitzgerald et al., 2006; Moharir et al., 2011). Venous infarction is common, and hemorrhagic venous infarction constitutes a large share of these lesions (Fitzgerald et al., 2006). Intracranial hemorrhage of some form is observed in many cases, whereas a truly “parenchyma-normal” presentation is relatively uncommon. These distributions themselves are instructive: in neonatal venous thrombosis, hemorrhage is frequent and may be viewed as part of the disease course, making it conceptually and empirically unsound to treat hemorrhage as an isolated prognostic penalty. A more coherent approach is to interpret hemorrhage within the spectrum of venous outflow–related parenchymal injury, as an outward marker that tissue damage has already occurred.

Registry data similarly support the view that parenchymal lesions are the rule rather than the exception. Among infants classified as having “isolated” venous thrombosis with analyzable imaging, only a minority lack parenchymal abnormalities, whereas most demonstrate venous ischemic infarction, hemorrhagic infarction or intracranial hemorrhage, or both (Bhatt and Chan, 2021). Even when outcome assessment is limited to discharge rather than long-term follow-up, such findings highlight a counseling-critical fact: many infants already carry a parenchymal injury burden at the moment venous thrombosis is diagnosed (Teixeira and Rosa, 2024). Bringing hemorrhage back into the venous injury spectrum therefore aligns better with the covarying evidence linking lesion burden to motor, language, cognition/behavior, and epilepsy risk.

### 8.3 Reframing within the HIE/NE context

Hemorrhage is particularly prone to becoming a confusing signal within hypoxic–ischemic encephalopathy cohorts because two qualitatively different categories often coexist. One is closer to perinatal or delivery-related processes and small-volume extra-axial bleeding. The other suggests impaired venous outflow or another vascular event. Data from hypothermia-treated cohorts provide an important calibration point: hemorrhage is detected in a substantial minority and is often small-volume, whereas long-term neurodevelopmental outcome is shaped predominantly by the burden of hypoxic–ischemic injury on MRI and spectroscopy; hemorrhage itself does not consistently show an independent effect. In typical hypothermia-treated cohorts, small-volume intracranial hemorrhage is therefore unlikely to represent a major change in prognosis (Lakatos et al., 2019; Lally et al., 2019).

A counseling-oriented stratification language can be organized into three steps. First, establish baseline prognosis using the burden and pattern of hypoxic–ischemic injury on structural imaging and spectroscopy (Perera et al., 2022). Second, assess whether venous system–related phenotypes are present–such as hemorrhagic venous infarction, intraventricular hemorrhage accompanied by unilateral thalamic or other deep hemorrhage, or lateralized parenchymal lesions that do not fit canonical hypoxic–ischemic distributions–and re-stratify baseline risk accordingly, because these phenotypes introduce the long-term risk profile associated with venous thrombosis (Ranjan et al., 2023). Third, translate that risk elevation into its most explanatory substrates: the extent of parenchymal injury, deep structure involvement, lesion evolution, and the likelihood of epilepsy, rather than escalating risk simply because “blood is seen” (Kalogeris et al., 2012). This logic is internally consistent with the evidence base emphasizing high rates of parenchymal lesions and meaningful long-term functional impairment and seizure risk, and it aligns with syntheses in which parenchymal injury is the dominant determinant of adverse outcomes.

Overall, the intent of this section is not to endorse slogans such as “hemorrhage equals harm” or “hemorrhage does not matter,” but to provide transferable causal and stratification language within the hypoxic–ischemic encephalopathy framework: first quantify injury burden, then identify venous significance, and finally anchor prognosis in tissue-level questions of how much injury, where it is located, and how it evolves. This approach more reliably aligns family counseling and follow-up cadence with the true risk landscape.

## 9 Research gaps and a methodological standardization agenda

### 9.1 A draft “minimum MRV reporting standard”: sequences, parameters, interpretive thresholds, and adjudication pathways

Within imaging pathways for neonatal encephalopathy and hypoxic–ischemic encephalopathy, first-line consensus around MRI is relatively well established, with T1/T2-weighted imaging, diffusion-weighted imaging, and susceptibility-weighted imaging forming the core, and angiographic or venographic sequences added when indicated (Halefoglu and Yousem, 2018). What continues to drive marked inter-center polarization in reported detection rates of cerebral venous sinus thrombosis is not MRI *per se*, but the variability in MRV technique and interpretation (Ruderman et al., 2025). Differences in technique (two- versus three-dimensional time-of-flight, phase-contrast, contrast-enhanced approaches), acquisition plane and key parameters, and reader thresholds for distinguishing normal variants or artifacts from pathology all contribute. For this reason, flow-related signal loss, anatomical variants, image-quality limitations, and parenchymal–venographic concordance should be recorded separately in standardized MRV reporting (Table 1). In neonates, slow flow, small sinus caliber, and in-plane saturation effects converge to make two-dimensional time-of-flight venography particularly prone to spurious defects, while the temporal dynamics of recanalization introduce an opposing risk of false negatives. Under conditions dominated by methodological noise, single-center reports of “high” or “low” incidence are inherently difficult to generalize.

The core purpose of a “minimum reporting standard” is therefore not to mandate venography for all cases, but to define a reusable minimal information set. Reports should explicitly state the venographic technique and key acquisition parameters, including imaging plane, slice thickness, echo and repetition times, flip angle, and whether contrast enhancement was used (Lombardi et al., 2021). Interpretation should be grounded primarily in source images, with maximum-intensity projections reserved for display. Bidirectional cross-checking against structural imaging and susceptibility-weighted imaging is required, and reports should explicitly document parenchymal findings that align with impaired venous outflow, such as venous infarction, hemorrhagic transformation, or intraventricular hemorrhage accompanied by unilateral thalamic or other deep hemorrhage. Anatomical variants and artifact patterns, including transverse sinus dominance or segmental narrowing, should be clearly distinguished. Diagnostic conclusions should be tiered (for example, definite, probable, or not supported), with predefined adjudication pathways. When time-of-flight findings are discordant with parenchymal phenotype or remain equivocal, adjudication should preferentially proceed to contrast-enhanced three-dimensional venography or CT venography, rather than relying on a single projection image. These proposed minimum elements are summarized as a draft reporting and adjudication framework (Table 2).

**TABLE 2:** Draft minimum MRV reporting standard and adjudication pathway.

Domain	Minimum elements to report	Operational notes
Indication/trigger	Phenotype or clinical scenario prompting MRV (e.g., IVH with unilateral deep hemorrhage; hemorrhagic lesion crossing arterial territories; marked lateralization; suspected stroke; post-rewarming symptom evolution).	Pre-specify triggers in protocols to reduce spectrum bias.
Timing	Postnatal day and relationship to cooling/rewarming; interval from symptom onset to imaging.	Timing affects DWI yield and may miss early recanalization.
Technique	MRV technique used (2D TOF, 3D TOF, PC-MRV, CE-MRV) and coil/field strength if relevant.	Technique choice changes artifact profile and sensitivity to slow flow.
Acquisition geometry	Acquisition plane, slab coverage, and anatomical extent (SSS, straight sinus, deep venous system, transverse/sigmoid sinuses, jugular bulbs).	Partial coverage can mimic segmental occlusion.
Key parameters	Slice thickness; TE/TR; flip angle; for PC-MRV, velocity encoding; for CE-MRV, contrast timing and bolus method.	Report parameters that influence in-plane saturation and signal dropout.
Reconstruction and reading basis	State whether source images were reviewed; MIP used for display only; any thin-slab MIP or reformatting.	Interpretation should not rely on a single MIP image.
Image quality	Motion/ghosting; saturation artifacts; signal-to-noise limitations; overall quality rating.	Document artifacts that could drive false positives/negatives.
Variant/artifact inventory	Transverse sinus dominance/hypoplasia; skull molding-related narrowing; common flow-gap locations and extent; in-plane saturation suspicion.	Report explicitly to avoid re-labeling variants as thrombosis.
Venous findings	Location/extent of non-visualization or stenosis; intraluminal signal if seen; collateral venous enlargement; deep venous involvement.	Describe laterality and segmental anatomy precisely.
Parenchymal concordance	Presence and laterality of venous-significant parenchymal phenotypes on structural imaging, diffusion, and susceptibility sequences.	Require lesion–venous congruence as a diagnostic constraint.
Diagnostic confidence	Tiered conclusion: definite/probable/indeterminate/not supported, with a brief rationale.	Facilitates cross-center comparability and downstream analyses.
Adjudication pathway	Predefined escalation when TOF findings are equivocal or discordant: CE-MRV, PC-MRV, or CTV; consider repeat imaging.	Avoid definitive labeling when evidence is incomplete.
Follow-up imaging	Planned re-imaging window; endpoints (recanalization, propagation, new lesions).	Tie imaging endpoints to clinical and functional follow-up.
Data availability	Specify whether de-identified source images and reading forms are stored for centralized review.	Enables blinded re-read and agreement assessment.

To enhance comparability across research and practice, multicenter studies should implement centralized blinded image review with formal agreement assessment, mandate reporting of image quality and artifact features (including the presence and extent of flow gaps in commonly affected sinus segments), and preferentially acquire MRI with venography in a unified post-rewarming window. Repeat imaging may be incorporated to capture temporal dimensions of thrombus formation, progression, and recanalization. At the study level, perinatal and postnatal covariates related to thrombotic risk and hypoxic–ischemic injury burden should be recorded concurrently to allow statistical separation of population and methodological confounding (Mota-Rojas et al., 2022). Only by standardizing how images are acquired, interpreted, adjudicated, and reported can detection rates and outcome effects of venous thrombosis move from center-specific experience to data that can be compared reliably across centers.

### 9.2 The relationship between therapeutic hypothermia and venous thrombosis: what remains to be proven

Whether therapeutic hypothermia modifies the risk of neonatal cerebral venous sinus thrombosis remains a question of association rather than causation. Existing reports are easily overinterpreted because three layers of confounding coexist (Zhou et al., 2025). More severely ill infants are more likely to undergo intensive imaging, introducing detection bias. The timing of thrombus formation is often uncertain, potentially occurring around birth or several days later (Schulman et al., 2024). Finally, large inter-center differences in venographic technique and interpretive thresholds readily convert normal variants or artifacts into apparent pathology (Ayanzen et al., 2000). When reports of high detection are juxtaposed with those approaching zero, the most defensible conclusion is not that hypothermia does or does not induce thrombosis, but that current designs are insufficient to disentangle these confounders.

Progressing from association to causation requires two changes. The first is imaging timing and methodology. A unified post-rewarming window should serve as the primary MRI time point, with venography acquired in parallel. Contrast-enhanced three-dimensional venography or CT venography should be specified as the upper-tier adjudication standard for equivocal cases. Interpretation must return to source images rather than rely on projections alone, and structural imaging and susceptibility-weighted phenotypes should serve as prerequisites for venographic inference (Halefoglu and Yousem, 2018). Second, clinical confounders must be captured and modeled systematically. Infection, dehydration, circulatory instability, and dynamic platelet and fibrinogen profiles should be incorporated into a shared data framework, alongside hypoxic–ischemic injury burden and monitoring intensity as key covariates (Badulescu et al., 2025). Analytically, time-dependent exposure models combined with repeat imaging can better describe trajectories of thrombus evolution, propagation, and recanalization, while reducing the risk of conflating more intensive surveillance with higher apparent disease incidence (Kersbergen et al., 2009). Only if exposure to hypothermia remains associated with higher thrombosis occurrence or progression under comparable illness severity and detection frameworks can an independent effect be credibly discussed. Until then, the most cautious and accurate framing is to treat hypothermia as part of the systemic context rather than assign it a singular causal role.

### 9.3 Making outcome research comparable across centers

In neonatal venous thrombosis research, cross-study synthesis of outcomes is challenging not because follow-up is lacking, but because follow-up instruments, time points, and endpoints are highly heterogeneous. Studies that provide stable anchors for clinical counseling tend to share more disciplined follow-up structures (Moharir et al., 2011; Yang et al., 2010). These include standardized assessments along a unified timeline, reporting developmental outcomes at 18–24 months and extending into the preschool and school-age periods to capture later-emerging language, executive, and behavioral difficulties. Epilepsy is tracked using consistent definitions as a distinct endpoint, rather than being subsumed under broad neurological impairment categories. Parallel clarity is required for imaging endpoints. Hemorrhagic burden and parenchymal injury burden should be distinguished rather than conflated, with explicit criteria for venous infarction, hemorrhagic transformation, deep structure involvement, and diffuse edema (Kersbergen et al., 2011b). These phenotypes should then be aligned directly with functional outcomes, instead of substituting the binary presence or absence of hemorrhage as a proxy.

What can realistically be adopted across centers is a compact but comprehensive core outcome set and data dictionary. Such a set need not be exhaustive, but it should cover key functional domains and time windows. At minimum, this includes a standardized developmental assessment in infancy, a higher-order language and cognitive evaluation around school entry, and long-term follow-up of epilepsy as an independent outcome (Brown et al., 2020). Imaging datasets should retain source sequences and interpretive records to allow centralized, blinded re-review. Analyses should routinely adjust for hypoxic–ischemic injury severity and follow-up intensity, and, where appropriate, employ methods such as propensity scoring or target trial emulation to reduce systematic bias driven by differential surveillance.

Only when these elements are standardized and transparently reported can outcome research in neonatal venous thrombosis progress from disparate descriptive accounts toward pooled, quantitative effect estimates. Such a foundation is essential for advancing mechanistic understanding and for designing and interpreting interventional studies. The next meaningful step for the field may therefore be the establishment of an internationally adoptable core outcome set, aligned with registries and clinical trials alike. It need not be complex, but it must be sufficiently broad to encompass critical domains and developmental windows, including standardized assessment at 18–24 months and extended preschool or school-age follow-up to capture late-emerging cognitive and behavioral difficulties, with epilepsy defined and tracked as a distinct long-term endpoint. Within this framework, research can advance from reporting incidence and outcomes toward generating comparable, actionable evidence.

## 10 Conclusion

In term and near-term neonatal encephalopathy, including hypoxic–ischemic encephalopathy (HIE), intracranial hemorrhage should not be treated as a homogeneous finding. Small-volume extra-axial hemorrhage is often nonspecific, whereas certain phenotypes carry greater etiologic and prognostic weight. Using venous system–related hemorrhage as a working concept may help distinguish background hemorrhagic findings from patterns more likely to reflect impaired venous outflow, particularly cerebral sinovenous thrombosis–related hemorrhagic venous infarction and, in term infants, intraventricular hemorrhage accompanied by unilateral thalamic or other deep hemorrhage consistent with venous drainage anatomy.

Neuroimaging is central to this distinction. Structural imaging, diffusion-weighted imaging, and susceptibility-weighted imaging provide the core information for identifying parenchymal injury and hemorrhagic burden, while venography is most informative when triggered by directional imaging features rather than applied routinely. Marked lateralization, hemorrhagic lesions crossing arterial territories, and the combination of intraventricular hemorrhage with unilateral thalamic or deep hemorrhage should prompt reconsideration of venous involvement. At the same time, neonatal MR venography must be interpreted cautiously because flow-related artifacts and developmental venous asymmetries can mimic thrombosis; diagnostic confidence is strengthened by parenchymal–venographic concordance and, when needed, adjudication with complementary imaging.

From a prognostic perspective, available evidence suggests that risk is driven more by associated venous-related parenchymal injury than by hemorrhage alone. This has direct implications for management, follow-up, and family counseling. Future studies should prioritize more consistent imaging acquisition and reporting, careful capture of HIE injury burden and relevant confounders, and longer-term outcome assessment that includes epilepsy and later-emerging neurodevelopmental deficits. A phenotype-oriented approach may therefore improve both bedside decision-making and the comparability of evidence across centers.

## Figures and Tables

**Table:** 

Abbreviation/term	Meaning	Notes in this review
APTT	Activated partial thromboplastin time	Routine coagulation assay.
BGT pattern	Basal ganglia–thalamus pattern	Canonical injury pattern in term/near-term HIE.
CE-MRV	Contrast-enhanced MR venography	Adjudication technique when TOF-MRV is equivocal.
CSVT	Cerebral sinovenous thrombosis	Thrombosis of dural venous sinuses and/or deep venous system.
CT	Computed tomography	Used here mainly in the context of venous adjudication.
CTV	CT venography	Second-modality adjudication for uncertain MRV.
DIC	Disseminated intravascular coagulation	Systemic coagulopathy phenotype that may occur after asphyxia.
DWI	Diffusion-weighted imaging	Most sensitive for acute injury; optimal window often post-rewarming.
Flow gap	Focal non-visualization on TOF-MRV	Often reflects artifact or slow flow in neonates; interpret with parenchymal concordance.
GRE	Gradient-recalled echo	Susceptibility-based sequence; SWI is preferred when available.
HIE	Hypoxic–ischemic encephalopathy	Most common cause of neonatal encephalopathy in term/near-term infants.
HVI	Hemorrhagic venous infarction	Key parenchymal correlate of impaired venous outflow.
ICH	Intracranial hemorrhage	Umbrella term for extra-axial and intraparenchymal bleeding patterns.
In-plane saturation	TOF-related saturation artifact	Occurs when flow is parallel to the acquisition plane.
IPH	Intraparenchymal hemorrhage	Parenchymal hemorrhage; may be venous-related in specific phenotypes.
IVH	Intraventricular hemorrhage	In term/near-term infants, may signal venous outflow pathology when combined with deep hemorrhage.
MIP	Maximum-intensity projection	For display; source images should anchor interpretation.
MRA	MR angiography	Adjunct when arterial stroke is suspected.
MRI	Magnetic resonance imaging	Primary imaging modality in the manuscript.
MRV	MR venography	Venous imaging; technique-dependent and artifact-prone in neonates.
NE	Neonatal encephalopathy	Clinical syndrome; etiologies include HIE and vascular events.
PC-MRV	Phase-contrast MR venography	Alternative venographic technique with different artifact profile.
PT	Prothrombin time	Routine coagulation assay.
Recanalization	Restoration of venous patency	May occur early; can contribute to false-negative imaging if delayed.
Propagation	Thrombus extension	Clinically relevant early event in CSVT.
ROTEM	Rotational thromboelastometry	Viscoelastic testing platform that can be performed under temperature-adjusted conditions.
SDH	Subdural hemorrhage	Common extra-axial hemorrhage; often background finding in term infants.
SAH	Subarachnoid hemorrhage	Extra-axial hemorrhage subtype.
SSS	Superior sagittal sinus	Posterior segment is a common location for TOF flow gaps.
SWI	Susceptibility-weighted imaging	Core sequence for hemorrhage burden and venous conspicuity.
TE/TR	Echo time/repetition time	Key acquisition parameters that should be reported for MRV.
TEG	Thromboelastography	Viscoelastic testing platform.
TH	Therapeutic hypothermia	Standard care for moderate-to-severe HIE; modifies hemostatic kinetics.
TOF-MRV	Time-of-flight MR venography	Non-contrast MRV technique; high artifact burden in neonates.
vWF	von Willebrand factor	Often elevated in neonates; part of developmental hemostasis.
Watershed pattern	Border-zone injury pattern	Canonical HIE pattern affecting cortical/subcortical regions.
